# Single-cell RNA landscape of the osteoimmunology microenvironment in periodontitis

**DOI:** 10.7150/thno.65694

**Published:** 2022-01-01

**Authors:** Yue Chen, Hua Wang, Qiudong Yang, Wenhua Zhao, Yuyi Chen, Qiaoqi Ni, Wenlei Li, Jiali Shi, Wei Zhang, Lu Li, Yan Xu, Hengwei Zhang, Dengshun Miao, Lianping Xing, Wen Sun

**Affiliations:** 1 Department of Basic Science of Stomatology, The Affiliated Stomatological Hospital of Nanjing Medical University, Nanjing, China; Jiangsu Province Key Laboratory of Oral Diseases, Nanjing, China; Jiangsu Province Engineering Research Center of Stomatological Translational Medicine, Nanjing, China.; 2 Department of Pathology and Laboratory Medicine and Center for Musculoskeletal Research, University of Rochester Medical Center, Rochester, NY, USA.; 3 State Key Laboratory of Reproductive Medicine, The Research Center for Bone and Stem Cells, Department of Anatomy, Histology and Embryology, Nanjing Medical University, Nanjing, China.

**Keywords:** Osteoimmunology, Periodontitis, Single-cell RNA-seq, Mesenchymal stem cells, alveolar bone

## Abstract

Single-cell RNA sequencing (scRNA-seq) enables specific profiling of cell populations at single-cell resolution. The osteoimmunology microenvironment in the occurrence and development of periodontitis remains poorly understood at the single-cell level. In this study, we used single-cell transcriptomics to comprehensively reveal the complexities of the molecular components and differences with counterparts residing in periodontal tissues.

**Methods:** We performed scRNA-seq to identify 51248 single cells from healthy controls (n=4), patients with severe chronic periodontitis (n=5), and patients with severe chronic periodontitis after initial periodontal therapy within 1 month (n=3). Uniform manifold approximation and projection (UMAP) were further conducted to explore the cellular composition of periodontal tissues. Pseudotime cell trajectory and RNA velocity analysis, combined with gene enrichment analysis were used to reveal the molecular pathways underlying cell fate decisions. CellPhoneDB were performed to identify ligand-receptor pairs among the major cell types in the osteoimmunology microenvironment of periodontal tissues.

**Results:** A cell atlas of the osteoimmunology microenvironment in periodontal tissues was characterized and included ten major cell types, such as fibroblasts, monocytic cells, endothelial cells, and T and B cells. The enrichment of *TNFRSF21^+^* fibroblasts with high expression of *CXCL1, CXCL2, CXCL5, CXCL6, CXCL13*, and* IL24* was detected in patients with periodontitis compared to healthy individuals. The fractions of *CD55^+^* mesenchymal stem cells (MSCs), *APOE^+^* pre-osteoblasts (pre-OBs), and *IBSP^+^* osteoblasts decreased significantly in response to initial periodontal therapy. In addition, *CXCL12*^+^ MSC-like pericytes could convert their identity into a pre-OB state during inflammatory responses even after initial periodontal therapy confirmed by single-cell trajectory. Moreover, we portrayed the distinct subtypes of monocytic cells and abundant endothelial cells significantly involved in the immune response. The heterogeneity of T and B cells in periodontal tissues was characterized. Finally, we mapped osteoblast/osteoclast differentiation mediators to their source cell populations by identifying ligand-receptor pairs and highlighted the effects of Ephrin-Eph signaling on bone regeneration after initial periodontal therapy.

**Conclusions:** Our analyses uncovered striking spatiotemporal dynamics in gene expression, population composition, and cell-cell interactions during periodontitis progression. These findings provide insights into the cellular and molecular underpinning of periodontal bone regeneration.

## Introduction

Chronic periodontitis is a common disease consisting of chronic inflammation of the periodontal tissues. It is a highly prevalent disease in humans, affecting about 20%-50% of the worldwide population 1, 2. Patients with chronic periodontitis often suffer from alveolar bone loss due to increased osteoclast (OC)-mediated bone erosion and decreased osteoblast (OB)-mediated bone formation in response to local inflammation 3. Chronic periodontitis can predispose individuals to systemic diseases such as cardiovascular disease, diabetes, and Alzheimer's disease, as well as gastrointestinal diseases and adverse pregnancy outcomes 4-6. The primary goal of periodontitis treatment is to control the infection and inflammation, halt the periodontal tissue destruction, and prevent alveolar bone loss. Removal of microbial biofilms and suppression of inflammation by initial periodontal therapy may block the periodontal tissue degradation; however, only limited regeneration of lost tissues occurs, especially the dental alveolar bone 7-9.

During periodontitis progression, a large number of immune cells activate and modulate the immune response by producing cytokines and growth factors that affect bone cells, such as osteoclast and osteoblast activity 10. Immune cells including macrophages 11, regulatory T cells (T_reg_s) 12, T_H_17 cells 13, memory B cells, and plasma cells 14 have established relevance in periodontitis pathogenesis. In 2000, the term 'osteoimmunology' was coined by Arron and Choi to highlight the interaction between the immune cells and bone cells, as well as the abnormal bone metabolism caused by an immune imbalance 15. Nowadays, osteoimmunology has opened the field of bone research to all fields dealing with chronic inflammatory diseases that are linked to bone loss, such as rheumatoid arthritis, inflammatory bowel diseases, and periodontitis 3, 16.

Single-cell RNA sequencing (scRNA-seq) enables specific profiling of cell populations at single-cell resolution, which greatly revolutionizes our ability to study the immune microenvironment 17. scRNA-seq will pave the road for understanding deregulated osteoimmune interactions and more specific targeting of cells participating in pathological bone loss 18. In this study, we used single-cell transcriptomics to comprehensively resolve the osteoimmunology microenvironment that is involved in periodontal tissues. We used periodontal tissues from human chronic periodontitis before and after initial periodontal therapy, as well as from clinically healthy controls. This allowed unbiased assessment of many heterogeneous cells at the single-cell level, hence revealing the complexities of the molecular components and differences with counterparts residing in periodontal tissues.

## Results

### scRNA-seq and cellular constitution of human periodontal tissues

**Figure 1:**
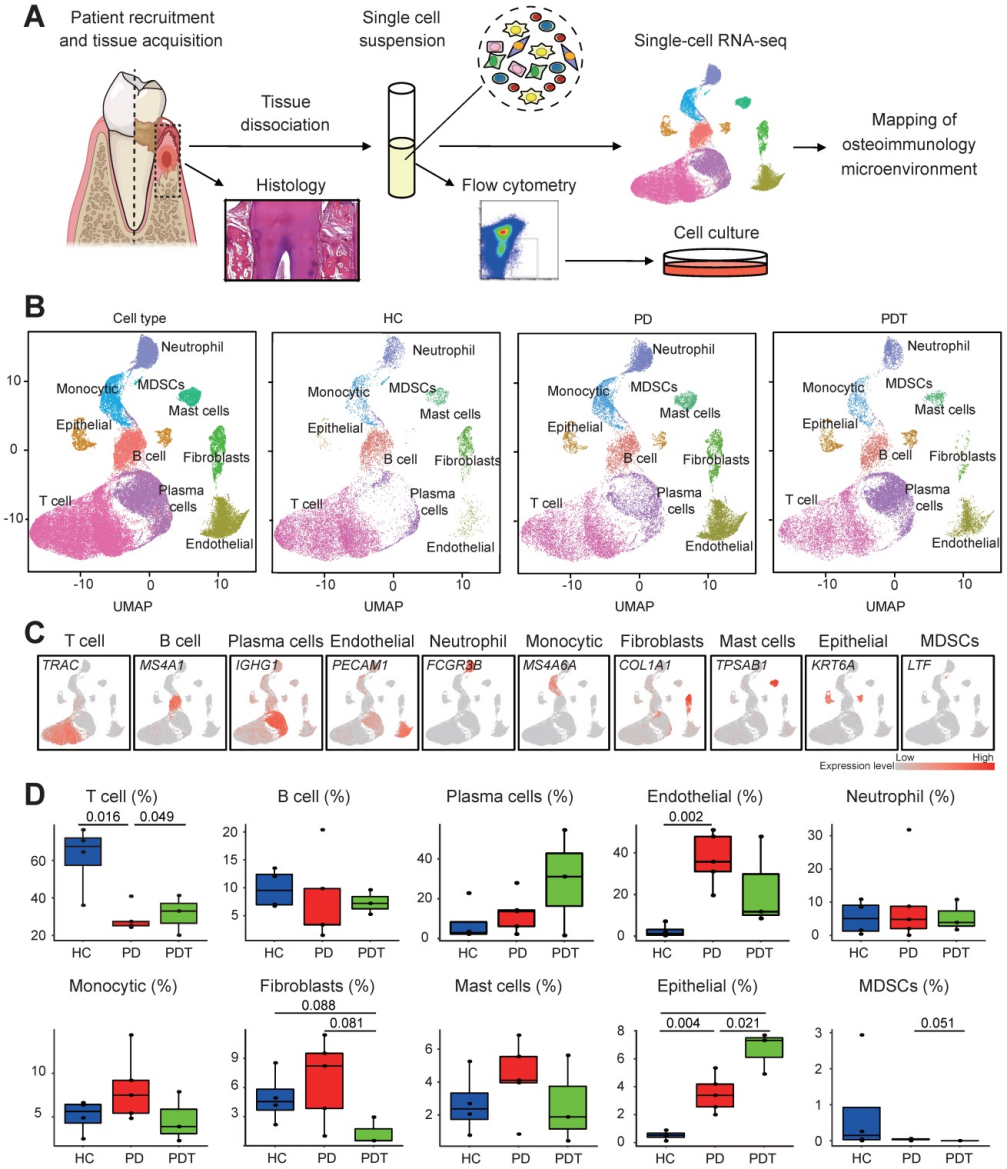
** Overview of the 51248 single cells from periodontal tissues of HCs, PDs, and PDTs. A.** Study overview. **B.** Uniform Manifold Approximation and Projection (UMAP) of the 51248 cells, colored by cell-type annotation from left to right: the total corresponding donors and the different conditions (HC, PD, and PDT). MDSCs: Myeloid-derived suppressor cells. **C.** UMAPs as in (B) but colored by expression of key cell-type marker genes. **D.** The box plots showing the percentage of cells for each of ten clusters as in (C) from HC (blue, n=14552), PD (red, n=19865), and PDT (green, n=16831) samples with plot center, box, whiskers and point corresponding to the median, IQR, 1.5 × IQR, and >1.5 × IQR respectively. Statistical analysis was performed using unpaired two-tailed t-tests.

To preliminarily probe the constitution of the cell populations in human periodontal tissues, we performed scRNA-seq analysis on human periodontal tissues from four healthy controls (HCs), five patients with severe chronic periodontitis (PDs), and three patients with severe chronic periodontitis after initial periodontal therapy within 1 month (PDTs) (Figure 1A). The clinical information of all the samples is shown in Table S1. After standard data processing and quality filtering (Method), we obtained single-cell transcriptomes from a total of 51248 single cells, including 14552 cells from HCs, 19865 cells from PDs, and 16831 cells from PDTs (Table S1).

Unbiased clustering of the cells identified ten clusters based on uniform manifold approximation and projection (UMAP) analyses. Each cluster was annotated based on the top principals and the marker genes were calculated (Figure 1B and Figure S1A). In particular, they were as follows: (1) T cell cluster, (2) B cell cluster, (3) plasma cell cluster, (4) endothelial cell cluster, (5) neutrophil cell cluster, (6) monocytic cell cluster, (7) fibroblast cell cluster, (8) mast cell cluster, (9) epithelial cell cluster, and (10) myeloid-derived suppressor cell cluster (MDSC) 19-22. The profiles of the expression differences of the representative marker genes in the cell populations were demonstrated by statistical quantification to match the biological annotation (Figure 1C, Figure S1B, and Table S2).

We next compared the proportion of each cell cluster in the different sample sets (Figure 1D). Of note, we observed significant reductions in the fraction of the T cell cluster in the PD group compared with the HC group, which was partly rescued after treatment (PDT group). The percentage of endothelial cells was clearly increased in the PD group compared with the HC group. Interestingly, the abundance of fibroblasts was significantly decreased in the PDT group compared with the PD group, which was even lower than that of the HC group. The percentage of epithelial cells was increased in the PD group compared with the HC group, which was even higher in the PDT group than the PD group. In addition, the distribution of MDSCs was almost specific to the HC group, which may be due to a very small amount of MDSCs causing variation (Figure 1D and Table S3).

### The heterogeneity of fibroblasts and the proinflammatory phenotype of* TNFRSF21*^+^ fibroblast subsets

**Figure 2:**
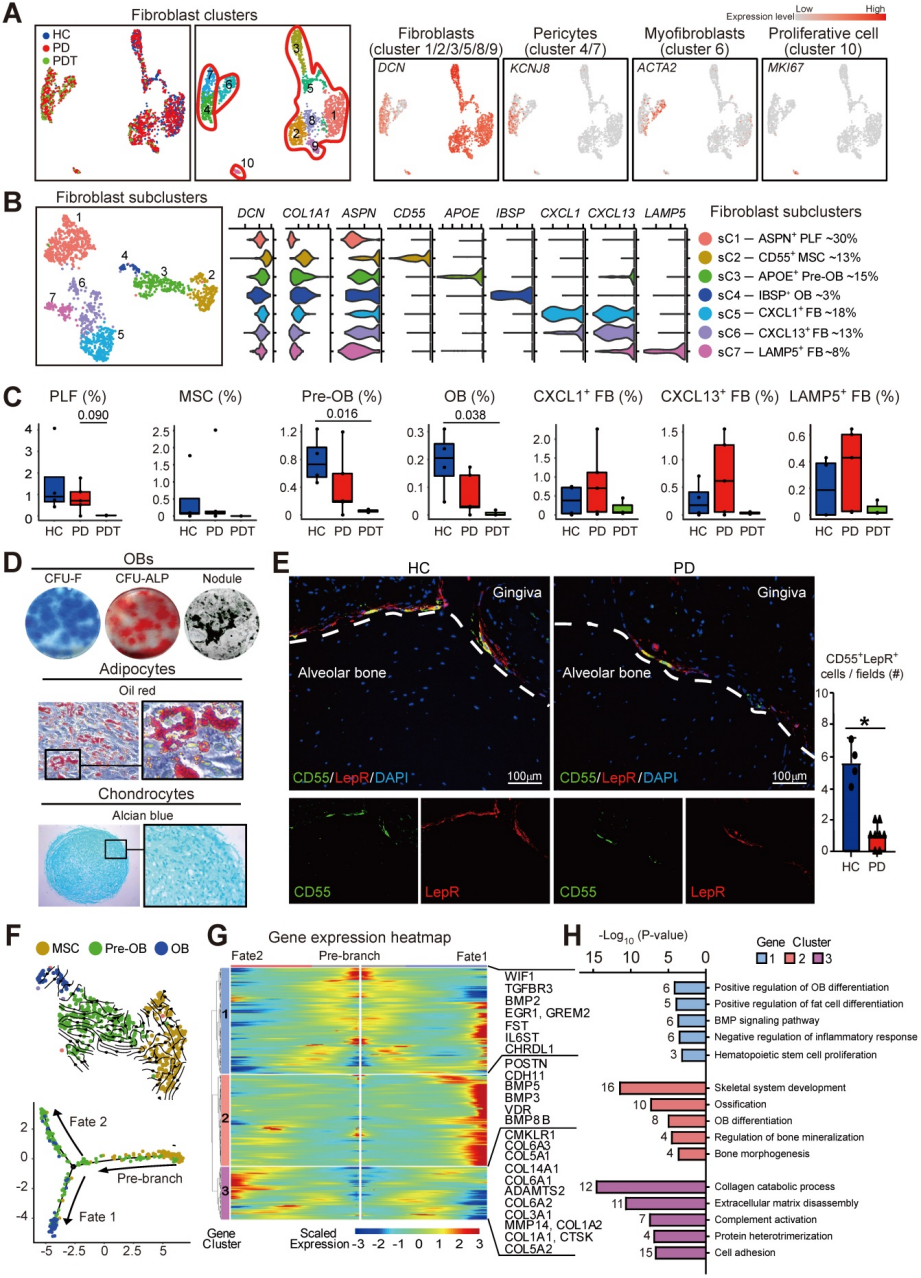
** Distinct subclusters of the osteoblastic lineage in fibroblast cells cluster. A.** Left panels: Uniform Manifold Approximation and Projection (UMAP) of 2194 fibroblasts (as in Figure 1A), annotated and colored by the sample type of origin (HCs, PDs, and PDTs) and clustering. Right panels: UMAPs color-coded for expression (gray to red) of key cell-type markers to define the clusters. Red contours (from left to right): Fibroblasts (cluster 1/2/3/5/8/9), Pericytes (cluster 4/7), Myofibroblasts (cluster 6), and Proliferative cell (cluster 10). **B.** Left panel: UMAP of 1358 fibroblasts (cluster 1/2/3/5/8/9 in A), annotated and colored by clustering. Center panels: violin plots showing distinct expressions of the selected marker genes (Row) in each subcluster. Right panel: identified subpopulations of Fibroblasts (subcluster 1-7) with the percentages shown. PLF: Periodontal Ligament Fibroblasts; MSC: Mesenchymal Stem Cell; pre-OB: pre-Osteoblast; OB: Osteoblast; FB: Fibroblast. **C.** The box plots showing the percentage of cells for each of seven subclusters as in (B) from HC (blue, n=655), PD (red, n=1095), and PDT samples (green, n=158) with plot center, box, whiskers, and points corresponding to the median, IQR, 1.5 × IQR and >1.5× IQR, respectively. Statistical analysis was performed using unpaired two-tailed t-tests. **D.** Representative images of *in vitro* assays to determine multiple differentiation potential of CD55^+^NT5E^+^LepR^+^ cells. Periodontal cells of a clinically healthy donor were collected and stained with various antibodies and subjected to flow cytometry sorting. CD55^+^NT5E^+^LepR^+^ cells were collected and subjected to the following assays. For osteogenic induction, colony-forming units (CFU-F, left)/alkaline phosphatase (ALP)-positive colony-forming units (CFU-ALP, middle)/nodules (right) formation were examined. For adipogenic induction, Oil red staining was performed to examine adipocytes. For chondrogenic induction, Alcian blue staining was performed to examine chondrocytes. **E.** Representative images of immunofluorescence staining (left panels) and the quantification (right panel) of periodontal tissues from HC (left) and PD samples (right) for double fluorescent analysis of CD55 (green) and LepR (red) expression. Top panel: Merged; bottom left: CD55; bottom right: LepR. Scale bar = 100μm. **F.** Upper panel: RNA velocity analysis of MSCs (subcluster 2), pre-OBs (subcluster 3), and OBs (subcluster 4) with velocity field projected onto the UMAP plot of fibroblast subclusters from Figure 2B. Arrows show the local average velocity evaluated on a regular grid and indicate the extrapolated future states of cells. Bottom panel: Monocle pseudotime analysis revealing the progression of MSCs (subcluster 2), pre-OBs (subcluster 3), and OBs (subcluster 4). Trajectory reconstruction of all single cells revealing three branches: Pre-branch, Fate 1, and Fate 2. **G.** Heatmap showing the scaled expression of differently expressed genes in three branches as in (F), cataloged into three major gene clusters (labels on left) that vary as a function of pseudotime, highlighting specific representative genes in each gene cluster along the right margin. From the center to the left of the heatmap, the kinetic curve from the pre-branch along the trajectory to fate 2 branch. From the center to the right, the curve from pre-branch to successful branch. **H.** GO analysis of differently expressed genes associated with three gene clusters as in (G) identified unique response pathways for each branch.

We further identified the cell populations of the fibroblast cluster by UMAP analyses. Fibroblast cells detected in Figure 1B were heterogeneous and clustered into four groups, including six clusters of fibroblasts (cluster 1/2/3/5/8/9), two clusters of pericytes (cluster 4/7), one cluster of myofibroblasts (cluster 6), and one cluster of proliferative cells (cluster 10) 23 (Figure 2A, Figure S2A, and Table S2). Concretely, the following cells were further identified from fibroblasts (cluster 1/2/3/5/8/9): four subclusters (sC) pertained to fibroblasts with unique phenotypes and functions (sC 1, 5, 6, 7) and three subclusters of osteoblastic lineage cells, including *CD55*^+^ MSCs (sC 2), *APOE*^+^ pre-OBs (sC 3), and *IBSP*^+^ OBs (sC 4) 24 (Figure 2B and Table S2).

A considerable amount of literature has been published to confirm the important roles of fibroblasts in regulating tissue homeostasis, coordinating inflammatory responses, and mediating tissue damage 25, 26. Four distinct fibroblast subclusters gained from Figure 2B are consistent with the theory that fibroblasts show different identities and have different roles in the extracellular matrix 27. Periodontal ligament fibroblasts (PLF, sC 1) occupied the most fibroblasts annotated by the expression of *ASPN*, an extracellular connective tissue marker intensely expressed within the PDL (Figure 2B and Figure S2B) 28. Intriguingly, three others of four subclusters expressed inflammatory-associated genes (*CXCL1, CXCL13, LAMP5*) (Figure 2B). An abundance of *CXCL1^+^, CXCL13^+^,* and *LAMP5^+^* subclusters was observed in periodontal tissues from PD samples (Figure 2C). Besides, *TNFRSF21,* which encodes CD358 protein and exerts crucial functions in inflammatory diseases 29, was detected in these three subclusters (Figure S2C). Immunofluorescence staining confirmed that collagen I^+^CD358^+^ fibroblasts were significantly increased in the periodontal tissues from PD samples compared with the HC samples (Figure S2D). Further, these *TNFRSF21^+^* subclusters also displayed high expression of genes involved in neutrophil trafficking factors (*CXCL1, CXCL2, CXCL5, CXCL6),* B cell and T_FH_ positioning (*CXCL13*), and upregulation of the immune system *(IL24)* (Figure S2E)*.* Therefore, *TNFRSF21^+^* fibroblast subsets might contribute to the activation of the proinflammatory transcriptome in periodontal tissue.

### Depletion of the osteoblastic lineage and failure of osteogenesis in response to initial periodontal therapy

In the osteoimmunology microenvironment, MSCs are characterized by a fibroblast-like morphology that can differentiate into a variety of cell types, including osteoblastic lineage cells 30. Subcluster 2, which was annotated as *CD55^+^* MSCs, also expressed other classical MSC markers, *NT5E* 31-33 and *LepR* 34, 35 (Figure S3A). We next used flow cytometry sorting to isolate CD55^+^NT5E^+^LepR^+^ or CD45^-^CD55^+^ cells from human periodontal tissues of clinically healthy donors. As expected, CD55^+^NT5E^+^LepR^+^ cells were shown to possess the characteristics of MSCs and exhibited *in vitro* multilineage differentiation potential (Figure 2D). Similar to CD55^+^NT5E^+^LepR^+^ cells, CD55 alone also could be used to label MSCs in human periodontal tissues. In contrast to CD45^-^CD55^-^ cells, CD45^-^CD55^+^ cells from periodontal tissues exhibited *in vitro* OB differentiation potential and further differentiated into osteoblast-like cells depositing mineralized matrix upon transplantation into immunodeficient mice (Figure S3B-C). Furthermore, the fractions of *CD55^+^* MSCs, *APOE^+^* pre-OBs, and *IBSP^+^* OBs decreased significantly in response to initial periodontal therapy (Figure 2C). Importantly, MSCs, pre-OBs, and OBs were almost depleted in the PDT group. We also confirmed the expression of CD55^+^LepR^+^ MSCs by immunofluorescence staining using periodontal tissues from HC and PD samples. As expected, the intense expression of CD55 on the surface of the LepR^+^ population was detected in periodontal tissues from HC samples, accompanied by relatively low expression of the CD55^+^LepR^+^ population in the PD samples (Figure 2E).

We further examined the impact of disease progression on osteoblastic differentiation in periodontal tissues. Because of the undetected MSCs in PDT, data from all participants were merged, and single-cell trajectories analysis was performed to model the lineage relationships among MSCs (sC 2), pre-OBs (sC 3), and OBs (sC 4). We utilized scVelo to determine the transcriptional fate of osteoblast-lineage cells. Projection of the velocity field arrows onto the UMAP plot extrapolated future states of CD55^+^ MSC to Pre-OB and OB population (Figure 2F). Monocle pseudotime analysis further corroborated the MSC to Pre-OB and OB transition. Interestingly, the pseudotime trajectory began with MSCs and then split into two main branches with OBs and pre-OBs placed at opposite divergent ends as two terminally differentiated cell types (Figure 2F). OBs were found at one terminal end of the trajectory, representing a successful transition from MSCs to OBs (fate 1 branch). In contrast, part of pre-OBs were found at another terminal end of the trajectory (fate 2 branch). With this in mind, we tried to elucidate the molecular dynamics that distinguished the two branches. The analyses of the gene expression dynamics revealed that along with fate 1 branch, cluster 2 genes activated at the end of the transition were predominantly involved in the GO terms “skeletal system development”, “ossification”, and “OB differentiation” (*POSTN, BMP3, BMP5,* and* BMP8B*) which are consistent with the features of osteoblastic differentiation. The fate 2 branch expressed higher levels of cluster 3 genes enriched for the GO terms “collagen catabolic process” (*CTSK, ADAMTS2*, and *COL6A2*) and “extracellular matrix disassembly” (*MMP14*, *COL3A1,* and *COL6A1*) (Figure 2G-H and Table S4). Therefore, these unique gene expression patterns characterized a successful OB differentiation path and a functional difference at the pre-OB subcluster.

### Multilineage differentiation capacity of pericytes

**Figure 3:**
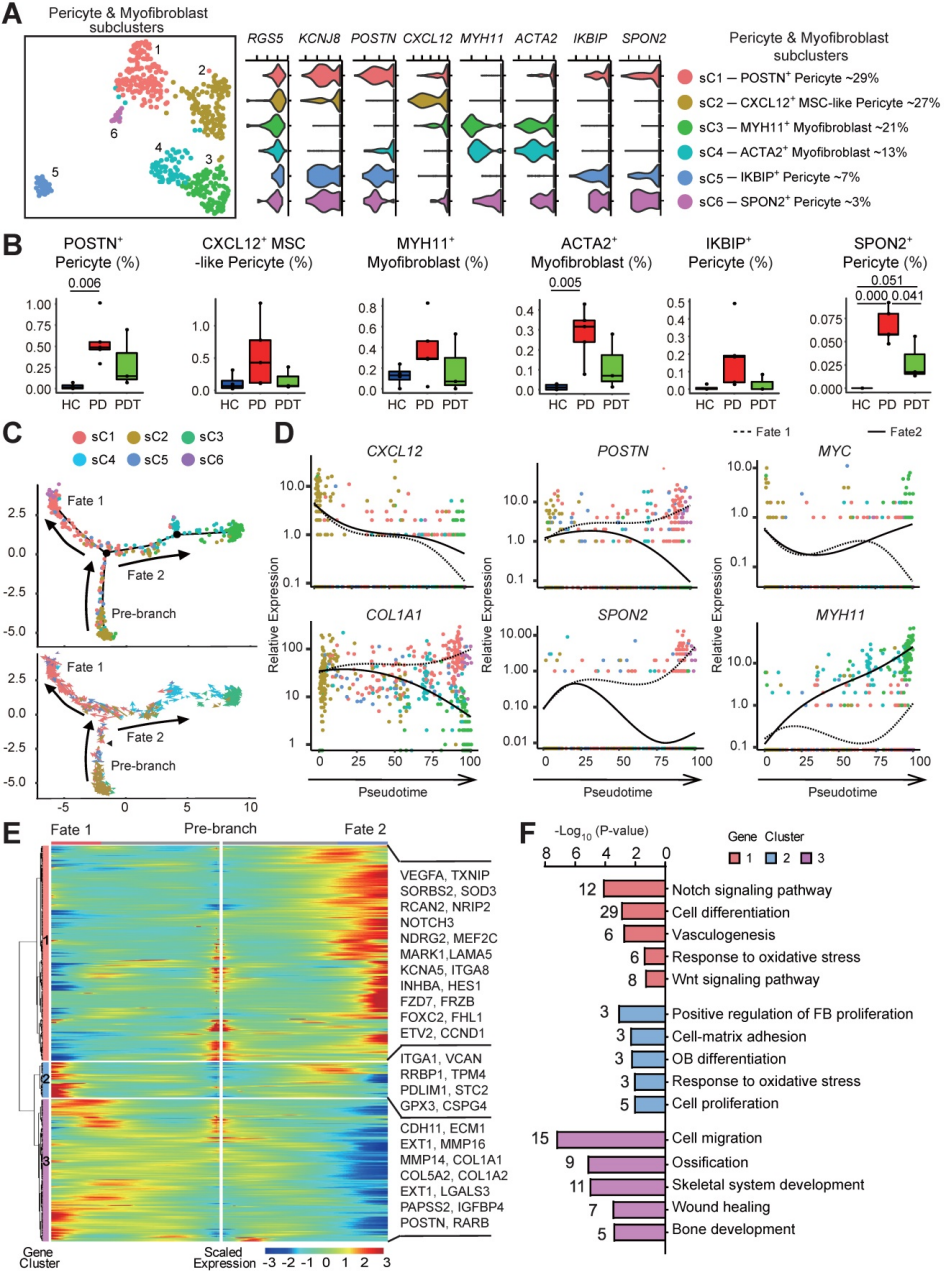
** The multilineage differentiation capacity of pericytes. A.** Left panel: UMAP of 521 cells as pericytes (cluster 4/7) and myofibroblasts (cluster 6) in Figure 2A, annotated and colored by clustering. Center panels: violin plots showing distinct expressions of the selected marker genes (Row) in each subcluster. Right panel: identified subpopulations of pericytes (subcluster 1-6) with the percentages shown. **B.** The box plots showing the percentage of cells for each of six subclusters as in (A) from HC (blue, n=42), PD (red, n=373), and PDT (green, n=106) samples with plot center, box, whiskers, and points corresponding to the median, IQR, 1.5 × IQR and >1.5× IQR, respectively. Statistical analysis was performed using unpaired two-tailed t-tests. **C.** Upper panel: Monocle pseudotime analysis revealing the progression of six subclusters as in (A). Trajectory reconstruction of all single cells revealing three branches: Pre-branch, Fate 1, and Fate 2. Bottom panel: RNA-Velocity analysis of the pseudotime trajectory of pericyte and myofibroblast. Direction of the arrows points to the cells are heading toward; length of the arrows reflects how fast the cells are heading toward a particular fate. **D.** The expression dynamics of selected marker genes go from *CXCL12^+^* MSC-like pericyte and bifurcating into two branches with respect to pseudotime coordinates. All single cells in the six subclusters are colored based on (A) and ordered based on pseudotime. **E.** Heatmap showing the scaled expression of differently expressed genes in three branches as in (C), cataloged into three major gene clusters (labels on left) that vary as a function of pseudotime, highlighting specific representative genes in each gene cluster along the right margin. From the center to the left of the heatmap, the kinetic curve from the root along the trajectory to Osteoblastogenesis. From the center to the right, the curve from the root to Myofibrogenesis. **F.** GO analysis of differently expressed genes associated with three gene clusters as in (E) identified unique response pathways for each branch.

Given the markedly decreased osteoblastic lineage cells with the absence of MSCs in periodontal tissues after initial periodontal therapy, we sought to explore other sources of MSCs. Pericytes safeguard vascular integrity as mural cells and possess attributes of self-renewal. As multipotent adult stem cells, they can commit and differentiate into multiple lineages for processes of tissue repair and regeneration 37-40. Notably, due to the paucity of distinctive markers 27, 41, single-cell data from pericytes and myofibroblasts were pooled and unsupervised clustering was conducted to identify distinguishable subpopulations. We noted that merged pericytes and myofibroblasts uniformly expressed *RGS5*, but the expression patterns and levels varied for individual canonical cell lineage markers (Figure 3A) 42. We detected two subpopulations of high *MYH11-* and *ACTA2-* expression myofibroblasts (subclusters 3 and 4). We also identified four transcriptionally distinct *KCNJ8^+^* pericyte subclusters. Among them, only sC 2 expressed a high level of *CXCL12*, a marker for perivascular MSCs 24. Hence, we identified this subcluster as *CXCL12^+^* MSC-like pericytes. Importantly,* CXCL12^+^* MSC-like pericytes existed even after initial periodontal therapy (Figure 3B and Table S3). Consistent with this, immunofluorescence staining showed that CXCL12^+^ cells are mainly located around the microvessels in periodontal tissues from HC group, whereas there was an increase in the expression of CXCL12^+^ cells with pericyte-like location in samples from PD group compared with that of HC group (Figure S4A). Interestingly, the* SPON2^+^* pericytes were clearly upregulated in the PD group compared with the HC group (Figure 3B and Table S3). The abundance of *SPON2^+^* pericytes with significantly high levels of cell proliferation-related genes (*MCM3*, *MCM4*, *MCM6*, *PCNA*) alluded that they were in a stimulated state (Figure 3A and Table S2).

To determine the lineage relationships and the corresponding gene expression, we performed pseudotime analysis on the merged cells to construct a new trajectory containing two termini corresponding to two distinct cell fates. The RNA velocity analysis was used to unambiguously demonstrate the trajectory (Figure 3C). CXCL12^+^ MSC-like pericyte was found at the start point of the trajectory, and then split into two main branches with *POSTN^+^*/*SPON2^+^* pericytes (fate 1 branch) and *MYH11^+^*/*ACTA2^+^* myofibroblasts (fate 2 branch) placed at opposite divergent ends as two terminally differentiated cell types. Of note, most CXCL12^+^ MSC-like pericytes failed to proceed through the commitment point (black triangles) and transition cells showed a propensity to go back to basal fate (Figure 3C). Indeed, differential gene expression analysis attributed the six clusters to the different subtypes concordant with the pseudotime states. Besides, we observed both cell fates enriched in the PD group and PDT group (Figure S4B). Next, we assessed the gene expression patterns responsible for the differentiation of MSC-like pericytes during the two cell fates. The expression of *CXCL12*, a critical hematopoietic stem cell niche factor in MSCs and pericytes 24, was found to be similar in both trajectories and was downregulated with cell differentiation. The expressions of *COL1A1*, *POSTN*, and *SPON2* were downregulated during fate 2 branch but notably upregulated during fate 1 branch (Figure 3D and Table S5).

The gene expression patterns involved in the continuum transition were further dissected. The cluster 1 genes, the expressions of which increased with fate 2 branch, were enriched for the GO terms “Notch signaling pathways”, “Wnt signaling pathways”, and pathways involved in “TGF-beta signaling pathways” (*FZD7, NOTCH3, INHBA*, and *MYC*) (Figure 3E-F and Table S5). These GO terms and pathways have also been reported during the process of fate 2 branch in renal interstitial fibrosis and pulmonary fibrosis 45-47. The cluster 2 and 3 genes exhibited branched-dependent enrichment in fate 1 branch and were involved in “osteoblast differentiation”, “ossification”, and “skeletal system development” (*MMP14*, *COL1A1*, *IGFBP4*, and *POSTN*) indicating the potential of osteogenesis (Figure 3D-F). Meanwhile, the gene clusters during both cell fates showed the same enrichment in the GO term “response to oxidative stress”, implying that inflammatory stimuli may contribute to both two important directions.

### The trajectory of osteoclast maturation in periodontal tissues

**Figure 4:**
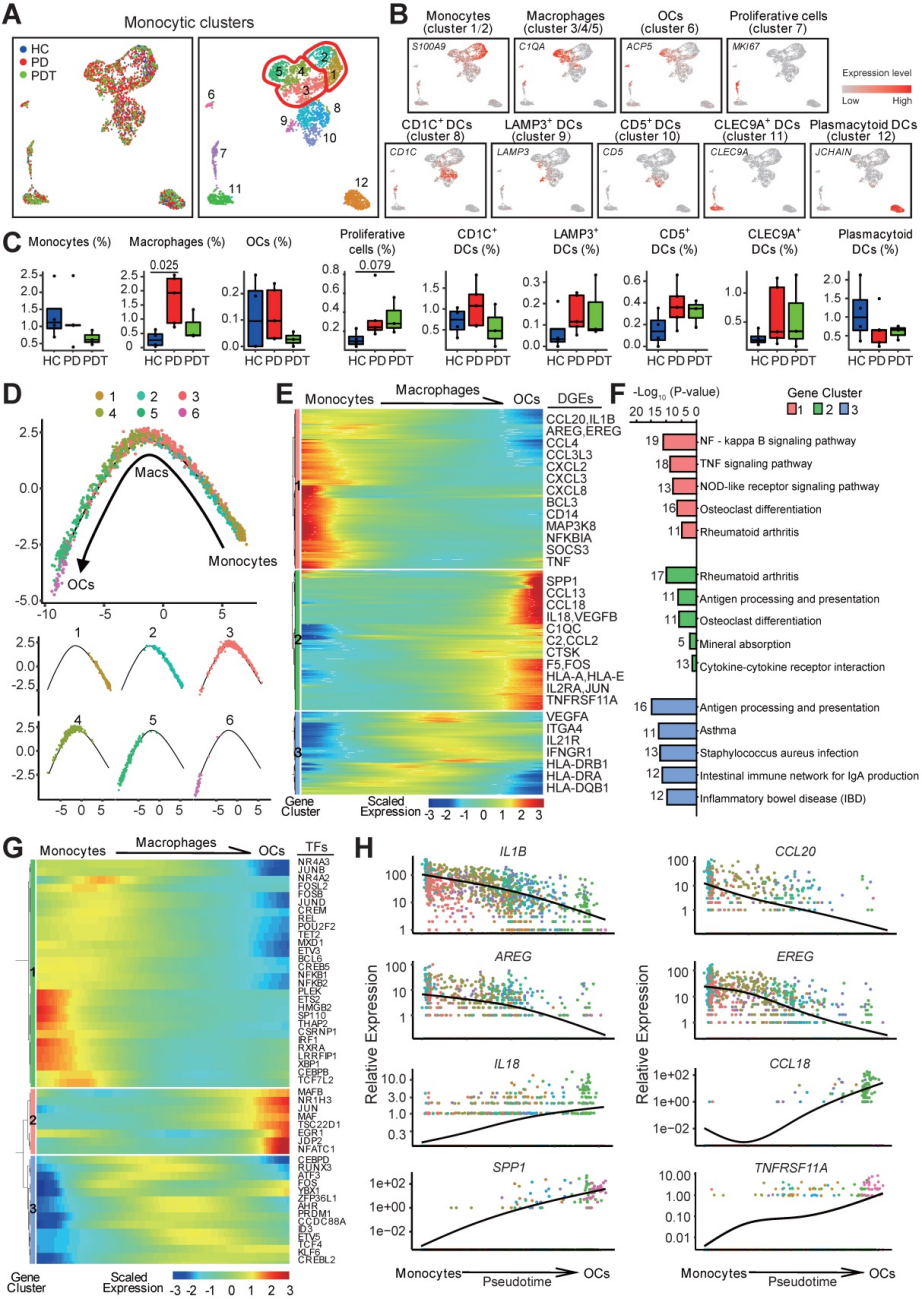
** Trajectory of the osteoclast maturation in periodontal tissues. A.** UMAP of 2874 monocytic clusters, annotated and colored by the sample type of origin (HCs, PDs, and PDTs) and clustering. Red contours: Macrophages (cluster 3, 4, 5; left); Monocytes (cluster 1, 2; right). **B.** UMAPs color-coded for expression (gray to red) of key cell-type markers to define the nine cell groups: Monocytes (cluster 1/2), Macrophages (cluster 3/4/5), OCs (cluster 6), Proliferative cells (cluster 7), CD1C^+^ DCs (cluster 8), LAMP3^+^ DCs (cluster 9), CD5^+^ DCs (cluster 10), CLEC9A^+^ DCs (cluster 11) and Plasmacytoid DCs (cluster 12). OCs: osteoclasts; DCs: Dendritic cells. **C.** The box plots showing the percentage of cells for each of nine groups as in (B) from HC (blue, n=627), PD (red, n=1577), and PDT samples (green, n=670) with plot center, box, whiskers, and points corresponding to the median, IQR, 1.5 × IQR and >1.5× IQR, respectively. Statistical analysis was performed using unpaired two-tailed t-tests. **D.** Monocle pseudotime analysis revealing the progression of three major groups: monocyte (cluster 1, 2), macrophage cell (cluster 3, 4, 5), and osteoclast (cluster 6). Trajectory reconstruction of all single cells revealing a continuous lineage path without a branch colored according to clusters as in (A) (top). The distribution of single cell from each cluster mapped in a continuous lineage path, respectively (bottom). **E.** Heatmap showing the scaled expression of differentially expressed genes in the cells of each major group as in (D), cataloged into three major gene clusters (labels on right) that vary as a function of pseudotime, highlighting representative differentially expressed genes in each gene cluster along the right margin. From the left to the right of the heatmap, a continuous lineage path from monocytes to OCs. OCs: osteoclasts; DEGs: differentially expressed genes. **F.** Pathway enrichment analysis associated with three gene clusters as in (E). **G.** Heatmap showing the scaled expression of differentially expressed transcription factor genes along with the pseudotime curve as in (D), cataloged into three major gene clusters (labels on right) that vary as a function of pseudotime, highlighting the top 50 transcription factors along the right margin. From the left to the right of the heatmap, a continuous lineage path from monocytes to OCs. OCs: osteoclasts; TFs: transcription factors. **H.** The expression dynamics of representative genes that encoded secretion protein. These genes are differentially expressed across pseudotime corresponding to three gene clusters as in (E). All single cells in the six subclusters are colored based on (A) and ordered based on pseudotime.

Monocytes, macrophages, OCs, and dendritic cells (DCs) are a closely related monocytic family that is characterized by its capacity to sense and respond to inflammation and bone damage. Its phagocytic properties and its high plasticity are controlled by the osteoimmunology microenvironmental heterogeneity 48. Within the monocytic cell cluster, we identified two clusters of monocytes (clusters1/2), three clusters of macrophages (clusters 3/4/5), one cluster of OCs (cluster 6), and the DC clusters with distinctive markers 49 (Figure 4A and Figure S5A). Groups were biologically annotated based on the expression of the cell-type marker genes (Figure 4B and Table S2). The fraction of macrophages was significantly increased in the PD group compared with the HC group but was partly rescued after initial periodontal therapy (Figure 4C). Similar changes were detected in the fraction of OCs but did not reach statistical significance. OC marker genes, including *ACP5, NFATC1,* and *TNFRSF11A*, were also slightly increased in the PD group compared with the HC group and were partly rescued after initial periodontal therapy (Figure S5B-C). Together, these data may suggest the efficacy of initial periodontal therapy in preventing additional bone loss by inhibiting the osteoclastic function.

To reveal the differentiation dynamics of the OC lineage cells, we reconstructed the developmental trajectory of monocytes (clusters 1/2), macrophages (clusters 3/4/5), and OCs (cluster 6). All the cells were contained within one cellular lineage without any bifurcations. Interestingly, three clusters of macrophages were found at different stages of differentiation, while cluster 5 was found near the terminal end of the trajectory (Figure 4D and Table S6). Besides, cluster 5 with high expression of osteoclast-related genes (*ACP5, CD14, FCGR3A,* and* ITGAV)* was proposed as osteoclast precursor cells (OCPs) (Figure 4A-B, Figure S5D, and Table S2). The gene expression patterns involved in the continuum transition were further dissected. Clusters 1 and 3 genes were repressed during osteoclastogenesis but were predominantly enriched in the inflammatory response pathways. Cluster 2 genes that activated at the final stage of osteoclastogenesis were enriched in the pathways about “OC differentiation” and “Mineral absorption” (Figure 4E-F). Interestingly, OCs possessed gene expressions related to the GO terms “immune response”, “antigen processing and presentation”, and “inflammatory response”, suggesting that OCs also contribute to inflammatory reactions (Figure S5E and Table S6).

Meanwhile, the transcription factors related to immune cell differentiation, such as *HMGB2*,* BCL6*, and *FOS*, were gradually downregulated along with the trajectory differentiation process (Figure 4G). Conversely, some osteoclast-related factors, such as *NFATC1, JUN, MAFB,* and *JDP2*, were upregulated in the process (Figure 4G). Furthermore, the expressions of *IL1B* and* CCL20* were highly expressed in monocytes/macrophages while decreased with osteoclastogenesis (Figure 4H). EGFR ligand genes* AREG* and *EREG* were overrepresented in monocytes/macrophages but decreased during osteoclastogenesis (Figure 4H), hinting that monocytes and macrophages may regulate the proliferation of epithelial cells, especially in an inflammatory status 50. Importantly, the expression of proinflammatory cytokines (*IL18* and *CCL18*) was highly expressed in OCPs, indicating that OCPs might also possess immune cell functions (Figure 4H and Table S4). As expected, the OC-related genes *SPP1* and* THFRSF11A* were enriched in OCs (Figure 4H).

### Abundant endothelial cells involved in the immune response

**Figure 5:**
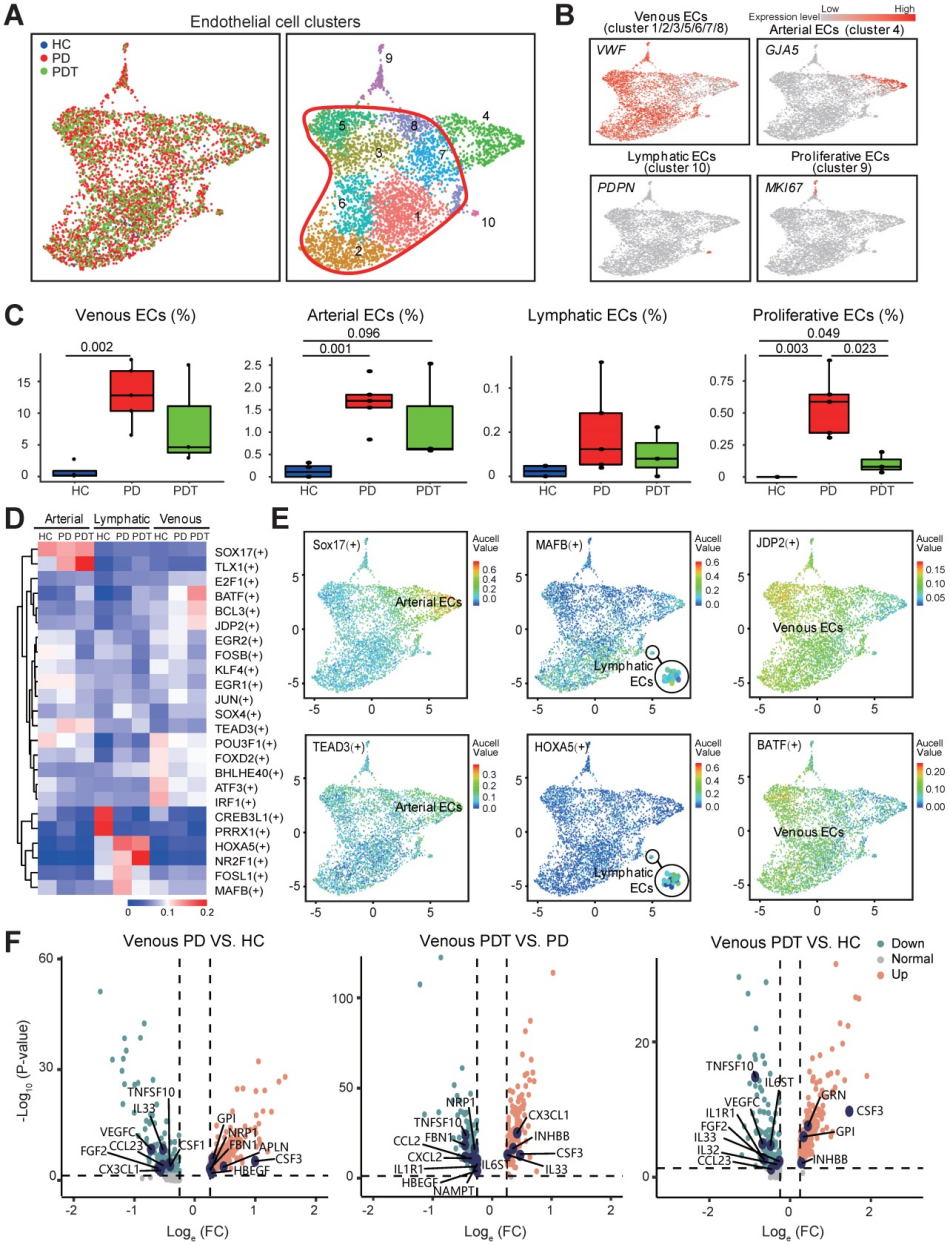
** Abundant endothelial cells involved in immune response. A.** UMAP of 4461 endothelial clusters, annotated and colored by the sample type of origin (HCs, PDs, and PDTs) and clustering. Red contour: Venous ECs (cluster 1/2/3/5/6/7/8). EC: Endothelial Cell. **B.** UMAPs color-coded for expression (gray to red) of key cell-type markers to define the four cell groups: Venous ECs (cluster 1/2/3/5/6/7/8), Arterial ECs (cluster 4), Lymphatic ECs (cluster 10), and Proliferative ECs (cluster 9). EC: Endothelial Cell. **C.** The box plots showing the percentage of cells for each of four cell groups as in (B) from HC (blue, n=153), PD (red, n=2999), and PDT samples (green, n=1309) with plot center, box, whiskers, and points corresponding to the median, IQR, 1.5 × IQR and >1.5× IQR, respectively. Statistical analysis was performed using unpaired two-tailed t-tests. **D.** Heatmap of the mean value of area under the recovery curve (Aucell) value of expression regulation by the transcription factors, as estimated using SCENIC, for the indicated three ECs clusters (Row). Shown the top 25 transcription factors genes (TF) (labels on right) having the highest difference in expression regulation estimates among HCs, PDs, and PDTs (Row). **E.** UMAP of endothelial cells, dots coded the for the Aucell value of the estimated regulon activity of selected TFs and the corresponding targeted genes in Arterial ECs (left), Lymphatic ECs (center), Venous ECs (right). EC: Endothelial Cell. **F.** Volcano plots displaying the differentially expressed genes in Venous ECs among PD VS. HC, PDT VS. PD, and PDT VS. HC (from left to right). Each dot represented one gene. Representative differentially expressed genes (blue) are indicated. Green dots, differentially down-regulated genes with log_e_FC < -0.25 and FDR < 0.05; red dots, differentially up-regulated genes with log_e_FC > 0.25 and FDR < 0.05; gray dots, non-differentially expressed genes.

Given the enhanced inflammatory infiltration, endothelial cells are abundant in periodontal lesions (Figure 1D). We identified ten transcriptionally distinct groups from 4461 endothelial cells and assigned them to known endothelial cell types 51 (Figure 5A and Figure S6A). The relative percentage of two distinct groups, venous endothelial cells (venous ECs, cluster 1/2/3/5/6/7/8) and arterial ECs (cluster 4), was higher in the PD group than in the HC group and was unchanged in the PDT group compared with the PD group. We also noticed a similar enrichment of lymphatic ECs (cluster 10) in the PD group, although no significant difference was detected (Figure 5A-C). The fraction of proliferative ECs was also increased significantly in the PD group compared with the HC group and was partly rescued after initial periodontal therapy (Figure 5C).

To examine the transcription factors contributing to the increased ECs in the PD samples, we applied single-cell regulatory network inference and clustering (SCENIC) analysis (Method) (Figure 5D-E). *SOX17* and *TEAD3*, transcription factors that may participate in arterial regeneration at homeostasis as well as in inflammatory conditions 52, 53, were potentially engaged in the changes of arterial ECs in the periodontal microenvironment (Figure 5D-E). We also found several unidentified regulators (*CREB3L1, PRRX1, MAFB,* and *HOXA5*) that were specifically expressed in lymphatic ECs. Expressions of both *CREB3L1* and* PRRX1* were downregulated in the PD samples compared with the HC samples. In contrast, the expressions of *MAFB* and *HOXA5* were in the opposite way. Of note, the AP-1 transcription factor family (*BATF, JDP2, JUN, FOSB,* and* ATF3*) was detected in venous ECs, which may mediate VEGF-induced endothelial cell migration and proliferation 54-57. Among them, *JPD2* and *BATF* were enriched in the PDT samples.

Subsequently, the expression of secretory factors was examined to explore the potential effects of venous ECs in the microenvironment (Table S7). Of note, the expression level of *CSF3* was upregulated in the comparisons of PD vs. HC, PDT vs. PD, and PDT vs. PD (Figure 5F and Table S7). Although CSF3 possesses a relatively low potency in inducing osteoclastogenesis compared with CSF1, CSF3 decreases bone mass by promoting OC function and inhibiting OB activity 58, 59. *CX3CL1* and* IL33*, cytokines that mediate the recruitment of immune cells 60-63, were both increased in the PDT group compared with the PD group. Furthermore, gene enrichment analysis (QuSAGE analysis, Method) revealed that compared with both the HC and PD groups, venous ECs in the PDT group were enriched by the immune response pathways, such as “antigen processing and presentation”, “Th1 and Th2 cell differentiation”, and “Th17 cell differentiation” (Figure S6B and Table S7). Therefore, these data may expand our understanding of the novel function of venous ECs in the periodontal microenvironment after initial periodontal therapy.

### The heterogeneity of T and B cells in the microenvironment of periodontal tissues

**Figure 6:**
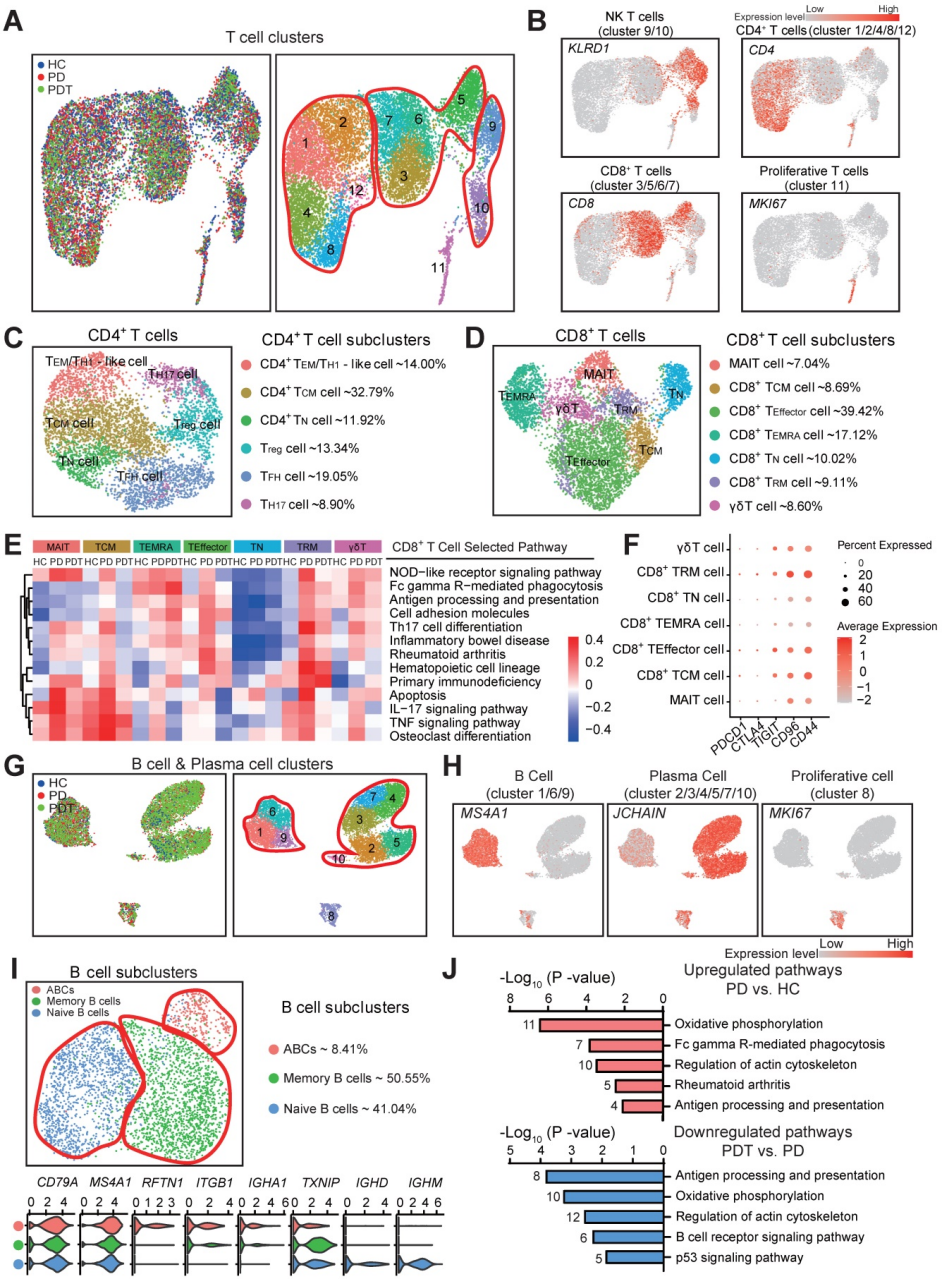
** The heterogeneity of T & B cells in periodontal microenvironment. A.** UMAP of 12805 T cell clusters, annotated and colored by the sample type of origin (HCs, PDs, and PDTs) and clustering. Red contours in right panel: CD4^+^ T cells (cluster 1/2/4/8/12) (left), CD8^+^ T cells (cluster 3/5/6/7) (center), NK T cells (cluster 9/10) (right). **B.** UMAPs color-coded for expression (gray to red) of key cell-type markers to define the four cell groups: CD4^+^ T cells (cluster 1/2/4/8/12), CD8^+^ T cells (cluster 3/5/6/7), NK T cells (cluster 9/10), Proliferative T cells (cluster 11). **C.** Left panel: UMAP of 5066 CD4^+^ T cells, annotated and colored by clustering with the label of each subcluster. Right panel: identified six subclusters of CD4^+^ T cells with the percentages shown. **D.** Left panel: UMAP of 5951 CD8^+^ T cells, annotated and colored by clustering with the label of each subcluster. Right panel: identified seven subclusters of CD8^+^ T cells with the percentages shown. **E.** Heatmap of the QuSAGE activity for the enrichment pathways (labels on the right) that associated with cell differentially expressed genes among seven subclusters of CD8^+^ T cells from HC, PD, and PDT samples (labels on the top). Red indicates an increased average expression of genes in the modules. **F.** Bubble plot showing expressions (dots) of the selected immune checkpoint inhibitor genes (columns) in each subcluster of CD8^+^ T cells (rows, as in B). Dot colored by the average expression level, and dot size proportional to the percentage expression. **G.** UMAP of 10576 B & Plasma cell clusters, annotated and colored by the sample type of origin (HCs, PDs, and PDTs) and clustering. Red contours: B Cell (cluster 1/6/9) (left), Plasma Cell (cluster 2/3/4/5/7/10) (right). **H.** UMAPs color-coded for expression (gray to red) of key cell-type markers to define the three cell groups: B Cell (cluster 1/6/9), Plasma Cell (cluster 2/3/4/5/7/10), and Proliferative cell (cluster 8). **I.** Top left panel: UMAP of 3248 B cells, annotated and colored by clustering. Red contours: Naive B cells (left), Memory B cells (center), ABCs (right). Top right panel: identified subclusters of B cells with the percentages shown. Bottom panel: Violin plots showing distinct expressions of the selected marker genes (row) in each subcluster (labels on left) of B cells. ABCs: Activated B Cells. **J.** Pathway enrichment analysis associated with genes upregulated (top) between PD vs. HC and downregulated (bottom) genes between PDT vs. PD.

The immune cells, especially T and B cells, are considered as important regulators in chronic inflammation 10. It was reported that plasma cells represent about 50% of cells in periodontitis lesions, while B cells comprise about 18%. The proportion of B cells was larger than that of the T cells in these lesions 64. However, in our study, the proportions of B cells and plasma cells were relatively lower than the T cells, but the amounts were considerable compared with other cell types (Table S3). Therefore, we further profiled and analyzed the T cells, B cells, and plasma cells in the microenvironment of periodontal tissues (Figure 6 and Figure S7-9).

Within the T cell clusters, we identified Natural killer T (NKT) cells, CD4^+^ T cells, and CD8^+^ T cells 65 (Figure 6A-B and Figure S7A). Consistent with prior studies 64, 66, seven subclusters of NKT cells with potential functional specificities were detected (Figure S7B-C). Next, six CD4^+^ T cell subclusters were identified (Figure 6C and Figure S8A), including CD4^+^ effector memory (T_EM_)/T_H1_-like cells (14%), CD4^+^ central memory T (T_CM_) cells (32.79%), CD4^+^ naïve T (T_N_) cells (11.92%), T_reg_ cells (13.34%), Follicular helper T (T_FH_) cells (19.05%), and T_H_17 cells (8.90%). Although these CD4^+^ T cell subclusters may perform distinct functions in the microenvironment of periodontal tissues, no statistical significance was detected during the periodontitis process (Figure S8B).

We further identified seven distinct CD8^+^ T cell subclusters (Figure 6D and Figure S8C), including mucosal-associated invariant T cells (MAIT, 7.04%), CD8^+^ T_CM_ cells (8.69%), CD8^+^ T_Effector_ cells (39.42%), CD8^+^ terminally differentiated effector T (T_EMRA_) cells (17.12%), CD8^+^ T_N_ cells (10.02%), CD8^+^ tissue-resident memory T (T_RM)_ cells (9.11%), and γδT cells (8.60%). Notably, with the reduction of T cells in the PD group compared to the HC group, CD8^+^ T_RM_ cells appeared to increase while MAIT and CD8^+^ T_EMRA_ cells significantly decreased in the PD group compared with the HC group (Figure 1D and Figure S8D). Interestingly, the gene set enrichment analysis revealed that various signaling pathways—including the NOD-like receptor signaling pathway, Th17 cell differentiation, apoptosis, IL-17 signaling pathway, TNF signaling pathway, and osteoclast differentiation, of most of the CD8^+^ T cell subclusters - were increased in the PD group compared with the HC group (Figure 6E). Most of the above pathways were partly rescued in the PDT group compared with the PD group (Figure 6E). In addition, both analyses about functional scoring and death inflammasomes of the CD8^+^ T cell subclusters showed that the gene expression signature patterns were in concordance with the enrichment of pathological pathways (Figure S8E and F). Besides, the key immune checkpoint genes (e.g., *PDCD1, CTLA4, TIGIT, CD96,* and *CD44*), which may induce an inhibitory response toward T-cell activation 67, 68, were detected in the CD8^+^ T cell subclusters (Figure 6F). Compared with *PDCD1* and *CTLA4* that have been previously reported 69, 70*,* the immune checkpoint genes *TIGIT, CD96,* and *CD44* were relatively upregulated in the CD8^+^ T cell subclusters.

Lastly, three transcriptionally distinct B cell subclusters were observed (Figure 6G-I and Figure S9A), including activated B cells (ABCs) (8.41%), memory B cells (50.55%), and naïve B cells (41.04%). To further identify the potential functions of the B cells in periodontitis, we performed differential expression analysis on the B cell expression profiles between PD and HC, PDT and HC, and between PD and PDT (Figure 6J and Figure S9C). The pathways in the PD group comprised of B cell functions (Fc gamma R-mediated phagocytosis, antigen processing and presentation, and regulation of actin cytoskeleton), and the inflammatory-specific signature (oxidative phosphorylation) was upregulated compared with the HC group. In addition, most pathways were rescued after initial periodontal therapy, but Fc gamma R-mediated phagocytosis was still overrepresented in PDT vs. PD, which may need further study in the future (Figure S9C and Table S8).

### Cell-cell communication in the periodontal osteoimmunology microenvironment

**Figure 7:**
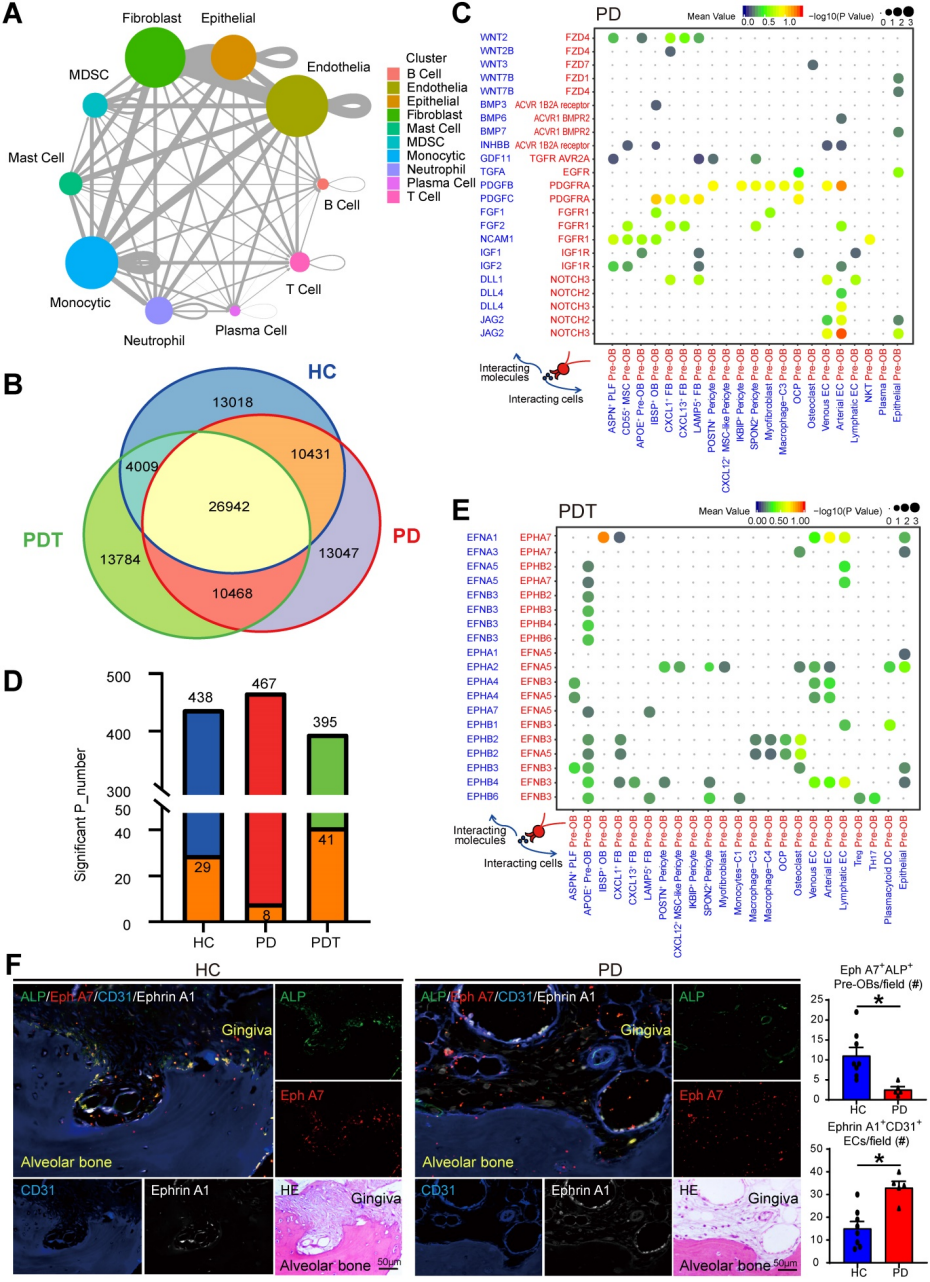
** Cell-cell communication among all cell types in the periodontal osteoimmunology microenvironment. A.** The circo plot showing the potential cell interactions among ten major cell types as in Figure 1B in periodontal tissues predicted by CellphoneDB. The node size represents the number of interactions. The width of the edge represents the number of significant ligand-receptor pairs between the two cell types. **B.** Venn diagram representing the interaction between the significant ligand-receptor pairs identified by CellPhone-DB analysis on scRNA-seq data from HC, PD, and PDT samples. **C.** The dot plot generated by CellPhoneDB showing potential ligand-receptor pairs associated with osteoblastogenesis between Pre-OB and all detected cellular types in PD group. Dots colored by mean expression of ligand-receptor pair between two clusters and dots size proportional to the value of -log10 (P Value). **D.** Stacked bar graph showing the number of all significantly expressed pairs between ECs and Pre-OB on different conditions (HC: blue bar; PD: red bar; PDT: green bar) and the Ephrin-EPH receptor pairs within these conditions (orange bar). **E.** The dot plot generated by CellPhoneDB showing potential ligand-receptor pairs associated with Ephrin-Eph receptor signaling pathway between Pre-OB and all detected cellular types in PDT group. Dots colored by mean expression of ligand-receptor pair between two clusters and dots size proportional to the value of -log10 (P Value). **F.** Representative images of paraffin sections from periodontal tissues of HC (left panel) and PD samples (right panel) were subjected to IF with anti-ALP (pre-OBs, green), anti-Eph A7 (red), anti-CD31 (ECs, blue), and anti-Ephrin A1 (white) Abs. Adjacent paraffin sections were stained with H & E staining to show the location. Top left: merged; top right: ALP; middle right: Eph A7; bottom left: CD31; bottom middle: Ephrin A1; bottom right: H & E. Scale bar = 50 µm. The quantifications of Eph A7^+^ALP^+^ Pre-OBs and Ephrin A1^+^CD31^+^ ECs per field were shown. Pre-OB: Pre-osteoblast, EC: Endothelial cell.

Elucidating the explicit interaction between bone cells and immune cells in the osteoimmunology microenvironment will shed light on the mechanisms of bone homeostasis and the pathogenesis of inflammatory osteolysis in periodontitis 3, 10. Therefore, we used CellPhoneDB (Method) to identify ligand-receptor pairs among the major cell types shown in Figure 1 to explore possible molecular interactions. The circos plot detected broadcast ligands and demonstrated extensive communication for cognate receptors (Figure 7A and Figure S10). Notably, endothelial cells showed the most interactions with other cell types, followed by fibroblasts, monocytic cells, and epithelial cells. Subsequently, we compared the ligand-receptor interaction pairs among the HC, PD, and PDT groups. In total, we identified 91699 ligand-receptor interaction pairs in samples from the HC, PD, and PDT groups. Interestingly, there were 26942 pairs in common among the three groups. There were 13018, 13047, and 13784 unique ligand-receptor interaction pairs in the HC, PD, and PDT groups, respectively (Figure 7B). Since pre-OBs and OCPs give rise to osteoblasts and osteoclasts, respectively, we further calculated the attraction strengths of ligand-receptor pairs in our scRNA-seq dataset and identified interaction pairs displaying significant cell population specificity between pre-OBs/OCPs and other cells.

The most specific interactions between pre-OBs and other cell types were observed with osteoblastogenesis (such as Wnt, BMP, PDGF, FGF, and NOTCH signaling), and were more abundant in the PD group than the HC group or PDT group (Figure 7C and Figure S11). Notably, pre-OBs in the PD group expressed relatively high levels of the NOTCH receptors, while the corresponding ligands were widely expressed in endothelial cells, suggesting a strong interaction between pre-OBs and ECs during the osteoblastogenic process 71. In addition to those commonly observed interactions, the receptor-ligand pairs enriched in Ephrin-Eph signaling that has rarely been reported in periodontitis were also detected. CellPhoneDB analyses showed apparently increased interactions of receptor-ligand pairs associated with Ephrin-Eph signaling between ECs and Pre-OB, such as EFNA1-EPHA7, EPHA2-EFNA5, EPHA4-EFNB3, EPHA4-EFNA5, and EPHB4-EFNB3 (Figure 7D-E, Figure S12, and Table S9). We next examined the anatomic relationship between Ephrin A1-expressing ECs (CD31^+^) and Eph A7-expressing Pre-OBs (ALP^+^) in periodontal tissues from HC and PD samples, using quadruple immunofluorescence (IF) staining on paraffin section. We observed numerous Eph A7^+^ALP^+^ Pre-OBs on alveolar bone surfaces in samples from HC group. In contrast, Eph A7^+^ALP^+^ Pre-OBs numbers in PD samples were markedly reduced, and numerous Ephrin A1-expressing ECs were detected (Figure 7F).

Finally, interactions involved in osteoclastogenesis between OCPs and other cell types, such as interactions via *TNFRSF11A, TNFRSF14, CSF1R, CSF3R,* and *NOTCH* receptors, were also changed among the three groups (Figure S13). For example, *TNFSF14* was expressed mainly by T cells, while the interaction of *TNFSF14-TNFRSF14* was only detected in the PD group. CSF3 released by venous ECs, as described above, was received by OCPs in the PD group. In addition, we observed cytokines and chemokines, such as *IL34*, *CCL8*, and *CCL19*, that were expressed mainly by pre-OB-like pericytes and received by OCPs in the PD group, while OCPs in the PDT group would receive stronger recruitment of *IL34* from pre-OBs and pre-OB-like pericytes through its receptor* CSF1R*. These data suggested that there were still active osteoclastogenesis-driven signals in the periodontal osteoimmunology microenvironment even after initial periodontal therapy, which may need more attention in further study.

**Figure 8:**
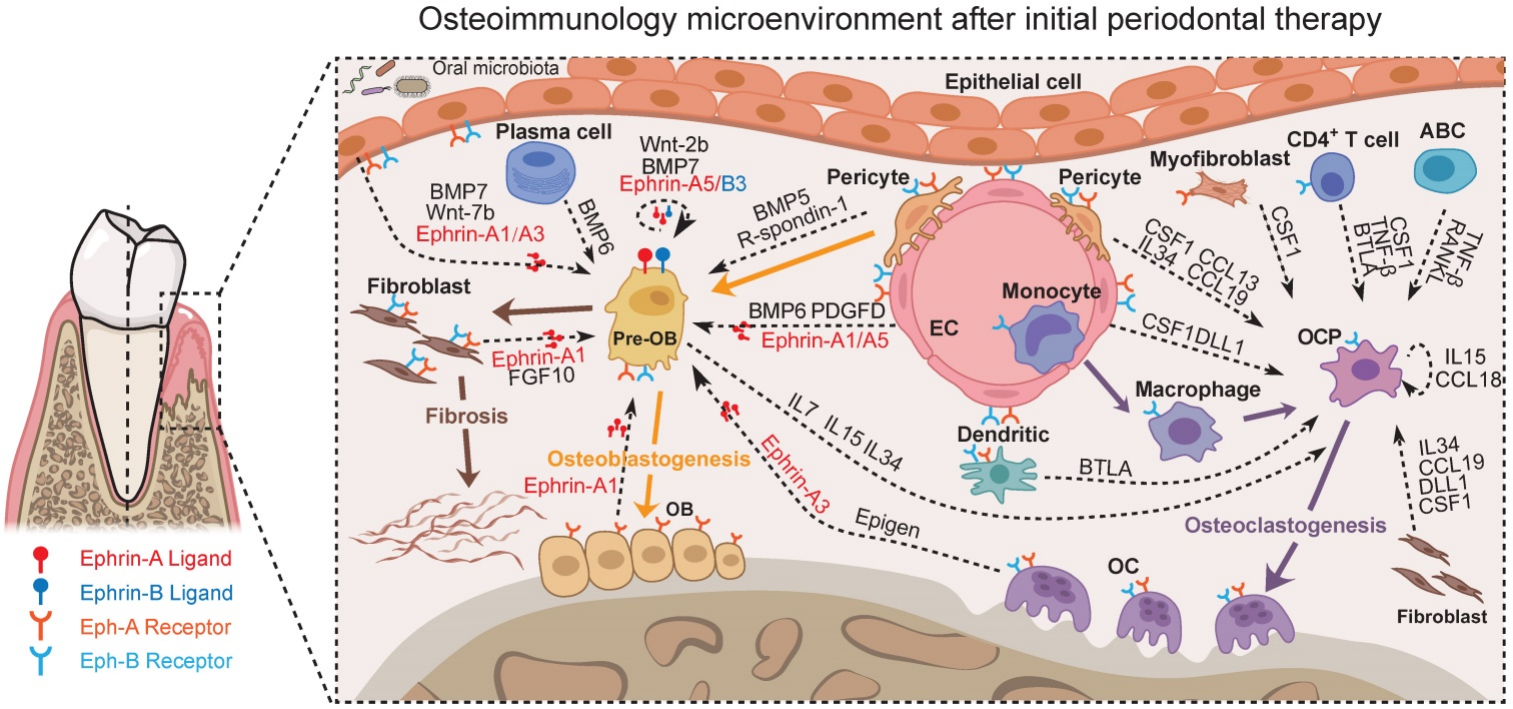
** Predicted regulatory network centered on the osteoimmunology microenvironment of periodontal tissue after initial periodontal therapy.** Sc-RNA seq analysis of the osteoimmunology microenvironment of periodontal tissue after initial periodontal therapy. In this Figure, the Ephrin-Eph signaling and the interactions regarding OB/OC differentiation are shown at the level of intercellular interactions. Pre-OB: Pre-osteoblast; OB: Osteoblast; EC: Endothelial cell; OC: Osteoclast; OCP: Osteoclast precursor cell.

Together, potential cross-talks with the focus on the osteoimmunology microenvironment of periodontal tissue after initial periodontal therapy could be abstracted from our data (Figure 7, Figure S10-13, and Table S9) as exemplified in Figure 8. These scRNA-seq data were direct evidence for a shared regulatory network among osteoimmune cells in periodontal tissue after initial periodontal therapy.

## Discussion

The inflammatory process of periodontitis causes the distinctive symptom of bone loss and a failure to return to homeostasis through excessive OC activation and the negative control of OB-mediated bone formation 72, 73. Therefore, it is important to achieve more understanding of the interplay between bone cells and immune cells. scRNA-seq approaches are rapidly becoming useful tools for deciphering both the abundance and functional state of immune cells 41, 74, 75, and have provided unprecedented detail of the heterogeneity of bone cells in bone-related diseases such as rheumatoid arthritis and osteoporosis 76, 77. While these scRNA-seq studies investigated only unilateral changes of either bone cells or immune cells, the robust evidence of how interactions of cellular components act on bone remodeling is still lacking, especially in periodontitis. Here, in the current study, scRNA-seq analysis was used to decipher the phenotypic and functional diversity of periodontal cells, and provide a better understanding of deregulated osteoimmune interactions participating in pathological bone loss.

CD55 is a glycosylphosphatidylinositol (GPI) -anchored surface glycoprotein that is widely distributed on blood, stroma, epithelial, and endothelial cells. The physiologic role of CD55 is to inhibit the complement cascade at the level of the critical C3 convertase step. By this mechanism, DAF protects autologous cells and tissues from complement-mediated damage and thereby plays a role in preventing or modulating autoimmune disease and inflammation. We also confirmed the wide expression of *CD55* by UMAP analysis (Date not shown). However, a couple of reports showed that MSCs also express CD55 78-81. In the current study, we characterized CD45^-^CD55^+^ cells as MSC population in periodontal tissue. CD45^-^CD55^+^ cells from periodontal tissues especially from alveolar bone exhibited *in vitro* OB differentiation potential and further differentiated into osteoblast-like cells depositing mineralized matrix upon transplantation into immunodeficient mice (Figure S3B-C).

Bone regeneration is in great demand nowadays for alveolar bone loss caused by periodontitis. However, our study demonstrates that the fractions of MSCs, pre-OBs, and OBs were significantly decreased in the PDT group compared with the PD group. Importantly, in the PDT group, we failed to detect MSCs (Figure 2 and Table S3). Such results replicated in three PDT samples were somewhat surprising but in keeping with the fact that clinical nonsurgical treatments for periodontitis have shown little bone regeneration 82. This raises an important question of how to promote efficient and effective periodontal bone regeneration without the help of MSCs. A variety of techniques, including the implantation of bone grafts or bone substitutes, root surface demineralization procedures, guided tissue regeneration, and the use of growth/differentiation factors, enamel matrix proteins, or various combinations thereof have been employed to achieve periodontal bone regeneration. However, limited bone regeneration has been achieved 82-84. Therefore, it is still urgent to find out other sources of MSCs after initial periodontal therapy. Our findings reveal that *CXCL12^+^* MSC-like pericytes have the potential of osteogenesis during inflammatory responses and may contribute to skeletal regeneration, even after initial periodontal therapy. These findings confirmed previous reports that injury-induced cues drive pericytes into the osteoblastic lineage with global upregulation of osteoblast-signature genes 37, 85. Therefore, pericytes may be a promising therapeutic candidate for treating bone loss in periodontitis after initial periodontal therapy.

The Eph receptors, the largest family of tyrosine kinase receptors, have been identified as playing important roles in a multitude of physiological and pathological activities, including bone remodeling, tissue repair, and fibrosis 86, 87. These membrane-bound molecules mediate contact-dependent and bidirectional signaling through both the Eph receptors (termed forward signaling) and ephrin ligands (referred to as reverse signaling) 88. In our study, the pronounced changes in the expression of Ephrin-Eph interactions indicated that they may play distinct roles in modulating the process of periodontitis (Figure 7, Figure S13, and Table S9). Evidence suggests that ephrin ligands and Eph receptors are crucial signaling molecules, contributing to fibroblast activation, extracellular matrix deposition, and tissue fibrosis formation 89-92. Pre-OBs have been described as one source of fibroblasts 93, 94. This shows diversity in origin beyond the local fibroblasts in organ fibrosis 95, 96. In our findings, the existence of pre-OBs but with failed osteogenesis in PDT may be associated with the altered cell fate toward fibroblasts (Figure 2, Figure 7, and Figure S12). Actually, tissue fibrosis and abnormal tissue remodeling have been described in periodontitis and lead to an imbalance in periodontal homeostasis 97-99. We assume that the abundance of Ephrin-Eph interactions in periodontal tissues from patients with severe chronic periodontitis after treatment has a significant influence on osteogenesis. This effect could be through both direct effects on osteoblast precursors and indirect effects via consuming the osteogenic lineage by enhancing fibrosis. Therefore, since the Eph receptors are potential therapeutic targets for multiple clinical conditions 90, 100-103, we speculate that the inhibition of Ephrin-Eph signaling may facilitate clinical bone defect healing after initial periodontal therapy.

Our data suggest enrichment of ECs in the inflammatory state of the PD group and the maintenance of ECs after treatment in periodontal tissues (Figure 5 and Table S3). These changes are consistent with the EC reactions in rheumatoid arthritis, suggesting ECs as active participants and regulators of the inflammatory process 61, 104, 105. We previously demonstrated the functions of lymphatic ECs in the pathogenesis of periodontal inflammation 106. The current study reveals the function of venous ECs in the recruitment of T cells via the production of proinflammatory cytokines and chemokines (Figure 5 and Figure S6). New research has revealed that ECs have many innate immune functions, including cytokine secretion, phagocytic function, antigen presentation, pathogen-associated molecular pattern- and danger-associated molecular pattern sensing, proinflammatory, immune-enhancing, anti-inflammatory, immunosuppression, migration, heterogeneity, and plasticity, suggesting ECs are novel immune cells 107-110. Along these lines, our data suggest ECs produce multiple proinflammatory cytokines/chemokines and possess the most interactions with other cell types (Figure 5, Figure 7, and Table S9). In addition, the interactions related to OB differentiation were most enriched between ECs and pre-OBs (Figure 7 and Table S9). Thus, we agree with the concept that ECs are dynamic cells that respond to extracellular environmental changes and play a meaningful role in immune system function. In the future, we may pay attention to the function of ECs in the inflammatory state in our research.

The activation of T and B cells is crucial in controlling chronic inflammation through constitutive cytokine secretion and modulation of osteoclastogenesis in the pathogenesis of periodontitis 111-113. The proportions of immune cell subtype in the current dataset appear at odds with previous studies in periodontal tissues 64, 66, 114, 115. The inconsistency was generated mainly for two reasons. The digestion condition optimized for bone cell collection in our study may not be the best condition for the yield of immune cells. On the other hand, the proportion of each cellular cluster in different sample sets shown in Figure 1D and Table S3 have suggested the variation among samples. We also observed a decline of MAIT cells in patients with periodontitis (Figure 6, Figure S8, and Table S3), raising the question of whether these cells might be related to an immune imbalance in periodontal tissues from patients with periodontitis. Further analyses revealed that signaling pathways, including NOD-like receptor signaling pathway, Th17 cell differentiation, apoptosis, IL-17 signaling pathway, TNF signaling pathway, and osteoclast differentiation, were activated in MAIT cells from patients with periodontitis compared with healthy individuals. This finding suggests the potential value of the dataset in selecting putative regulators of healthy versus disease MAIT states for further study.

Notably, periodontal tissue is a complex tissue composed mainly of alveolar bone, periodontal ligament, and gingival tissues. Conceptually, the present study would have benefitted from separate tissue collections to track tissue-specific cells in the subsequent analyses. However, if we separate bone, periodontal ligament, and gingiva during sample preparation for single-cell suspensions, multiple steps would cause the loss of cells and make the cells no longer fresh due to the prolonged exposure to artificial media and *in vitro* conditions. These unknown conditions may cause the inaccurate single-cell atlas of cellular populations and osteoimmune interactions in periodontal microenvironment. Another concern is that several cell types, such as osteocytes were undetected in fibroblast clusters (Figure 2), whereas melanocytes and merkel cells were undetected in the epithelial cell clusters (Figure S14). Indeed, the collection of osteocytes is quite challenging at this time due to their matrix-embedded anatomical location for scRNA-seq. As for melanocytes, there are a lot of factors that affect the number of melanocytes in the gingival epithelium, such as smoking, race, and ethnicity 116, 117. In addition, merkel cells have been proved that it is not easy to observe in normal or acute inflammation 118. As reported by Williams et al., melanocytes and merkel cells were found in a small proportion or even absent in human oral mucosa 66. Finally, similar to other single-cell studies 64, 66, our scRNA-seq analysis using the BD Rhapsody system also showed no difference in neutrophils across health and disease. Therefore, future studies will be aimed at solving these problems.

In summary, using scRNA-seq, we have provided a comprehensive portrait of the osteoimmunology microenvironment with multiple cell types and molecular mechanisms in periodontal tissue. The study identifies alterations in osteoimmune cell types that occur in the context of disease. The cells include fibroblasts, monocytic cells, endothelial cells, T cells and B cells. These results will enable other investigators to identify additional periodontitis-associated changes in a cell type specific manner for further study. This will likely accelerate mechanistic and functional investigations into the role of specific genes in relevant osteoimmune cell types and states in periodontitis.

## Materials and methods

### Patients

Detailed written informed consent was obtained from all volunteers in accordance with protocols approved by the Human Subjects Institutional Review Board of Nanjing Medical University (Approval ID NMU-2019313). All subjects were in good general health and had not taken anti-bacterial or anti-inflammatory drugs for 3 months before the sampling. Periodontal tissues, including alveolar bone, periodontal ligament, and gingival tissues, were obtained from subjects with clinically healthy periodontal tissues (HC), patients with severe chronic periodontitis (PD), and patients with severe chronic periodontitis after initial periodontal therapy within 1 month (PDT), as reported 119, 120. The alveolar bone that surrounds the roots of teeth is called the alveolar ridge of the jaw. Ridge repair is a common dental procedure often performed immediately following a tooth extraction. Periodontal tissues, including alveolar bone, periodontal ligament, and gingival tissues, were collected following tooth extraction and during ridge repair. 1) HC subjects (n = 4) showed no bleeding on probing, a probing depth < 3 mm, and no attachment loss or alveolar bone loss. Periodontal tissues from HC subjects were collected during teeth extractions for orthodontic reasons. 2) PD subjects (n=5) showed bleeding on probing, a probing pocket depth > 6 mm, and alveolar bone loss >60% of the root. Periodontal tissues from PD subjects were collected from teeth extracted after being judged irrational to treat. 3) PDT subjects (n = 3) showed negligible signs of marginal gingival inflammation but advanced clinical attachment loss and bone loss > 60% of the root. Periodontal tissues from PDT subjects were collected from teeth extracted after being judged irrational to treat. The collected periodontal tissue specimens were immediately placed in a sterile tube containing PBS and transferred to the laboratory within 10 min for single cell preparations and RNA sequencing.

### Preparation of single-cell suspensions

To harvest cells from periodontal tissues of human specimens, the tissues were minced on ice to pieces smaller than 1 mm^3^ and transferred to 5 ml of digestion medium containing 0.5 mg/ml collagenase type I (Sigma-Aldrich) and 0.5 mg/ml collagenase type II (Sigma-Aldrich) in PBS. Samples were incubated for 15 min at 37 °C, with manual shaking every 5 min. Samples were then vortexed for 10 s and pipetted up and down for 1 min using a 5 ml pipette. Next, 15 ml ice-cold PBS containing 0.04% BSA (Thermo Fisher Scientific) was added and samples were filtered using a 40 mm cell strainer (Thermo Fisher Scientific). The undigested alveolar bone fragments were collected and further digested using 0.5 mg/ml collagenase type I and 0.5 mg/ml collagenase type II at 37 °C for another 30 min to yield all the cells. After the supernatant was removed, the pelleted cells were suspended in red blood cell lysis buffer (Miltenyi Biotec) to lyse the red blood cells. After washing with PBS containing 0.04% BSA, the cell pellets were re-suspended in PBS containing 0.04% BSA and re-filtered through a 35 μm cell strainer. Dissociated single cells were then stained for viability assessment using calcein-AM (Thermo Fisher Scientific) and Draq7 (BD Biosciences). The single-cell suspension was further enriched with a MACS dead cell removal kit (Miltenyi Biotec).

### Single-cell RNA sequencing

A BD Rhapsody system was used to capture the transcriptomic information of the 12 sample-derived single cells. Single-cell capture was achieved by random distribution of a single-cell suspension across > 200,000 microwells through a limited dilution approach. Beads with oligonucleotide barcodes were added to saturation so that a bead was paired with a cell in a microwell. The cells were lysed in the microwell to hybridize the mRNA molecules to the barcoded capture oligos on the beads. Beads were collected into a single tube for reverse transcription and Exo I digestion. Upon cDNA synthesis, each cDNA molecule was tagged on the 5′ end (that is, the 3′ end of an mRNA transcript) with a unique molecular identifier (UMI) and cell barcode indicating its cell of origin. Whole transcriptome libraries were prepared using the BD Rhapsody single-cell whole-transcriptome amplification (WTA) workflow including random priming and extension (RPE), RPE amplification PCR, and WTA index PCR. The libraries were quantified using a high sensitivity DNA chip (Agilent) on a Bioanalyzer 2200 and the Qubit high-sensitivity DNA assay (Thermo Fisher Scientific). Sequencing was performed on an Illumina sequencer (Illumina) on a 150 bp paired-end run.

### Single-cell RNA statistical analysis

scRNA-seq data analysis was performed by NovelBio Bio-Pharm Technology Co. Ltd. with the NovelBrain cloud analysis platform. We applied fastp 121 with default parameter filtering the adaptor sequence and removed the low quality reads to achieve clean data. UMI-tools 122 was applied for single-cell transcriptome analysis to identify the cell barcode whitelist. The UMI-based clean data was mapped to the human genome (Ensemble version 91) utilizing STAR 123 mapping with customized parameters from the UMI-tools standard pipeline to obtain the UMI counts of each sample. The cells contained over 200 expressed genes and a mitochondrial UMI rate below 40% passed the cell quality filtering and mitochondrial genes were removed in the expression table. Seurat package (version: 2.3.4, https://satijalab.org/seurat/) was used for cell normalization and regression based on the expression table according to the UMI counts of each sample and the percent of mitochondria rate to obtain the scaled data. To remove the batch effect, which may affect the accuracy of single cell analysis, we applied the batch effect correction analysis by the Harmony package 124 based on the top 3000 variable genes with the default harmony parameter.

Utilizing the graph-based cluster method (resolution = 0.8), we acquired the unsupervised cell cluster result based the PCA top 10 principal. We calculated the marker genes by FindAllMarkers function with the Wilcox rank sum test algorithm under the following criteria: 1, lnFC > 0.25; 2, p-value < 0.05; and 3, min. pct > 0.1. In order to identify the cell type detailed, clusters of the same cell type were selected for re-tSNE analysis, graph-based clustering, and marker analysis. As for further re-cluster, we performed different resolutions to resolve subpopulations better according to the workflow as above.

### Pseudotime analysis

We applied the single-cell trajectories analysis utilizing Monocle2 (http://cole-trapnell-lab.github.io/monocle-release) using the DDR-Tree and default parameter. Before Monocle analysis, we selected marker genes from the Seurat clustering result and raw expression counts of the cell passed filtering. Based on the pseudotime analysis, branch expression analysis modeling (BEAM Analysis) was applied for branch fate determined gene analysis.

### Cell communication analysis

To enable a systematic analysis of cell-cell communication molecules, we applied cell communication analysis based on the CellPhoneDB 125, a public repository of ligands and receptors and their interactions. Membrane-secreted and peripheral proteins of the cluster of different time points were annotated. Significant mean and cell communication significance (p-value < 0.05) were calculated based on the interaction and the normalized cell matrix achieved by the Seurat normalization.

### SCENIC analysis

To assess transcription factor regulation strength, we applied the single-cell regulatory network inference and clustering (pySCENIC, v0.9.5) 126 workflow, using the 20-thousand motifs database for RcisTarget and GRNboost.

### QuSAGE analysis (gene enrichment analysis)

To characterize the relative activation of a given gene set, such as pathway activation, we performed QuSAGE 127 (2.16.1) analysis.

### Differential gene expression analysis

To identify differentially expressed genes among samples, the function FindMarkers with the Wilcox rank sum test algorithm was used under the following criteria: 1, lnFC > 0.25; 2, p-value <0.05; and 3, min. pct > 0.1.

### Co-regulated gene analysis

To discover the gene co-regulation network, find_gene_modules function of Monocle3 128 was used with the default parameters.

### Flow cytometry and sorting

Periodontal cells were harvested and red blood cells were lysed. Cells were stained with FITC-labeled anti-CD55 (catalog number MA1-19573, Invitrogen), eFluor 506-labeled anti-CD45 (catalog number 69-0459-42, Invitrogen), APC-labeled anti-NT5E (catalog number 17-0739-42, Invitrogen), and mouse anti-LepR (catalog number MAB867, R&D Systems) antibodies for 30 min, then incubated with PE-labeled anti-mouse antibody, and subjected to 11-colour LSRII (BD Biosciences) for cell sorting. CD55^+^NT5E^+^LepR^+^ or CD45^-^CD55^+^ cells were further subjected to cell culture as described next.

### Cell culture

(1) For CFU-F colony formation assays, cells were cultured at a density of <1 × 10^3^ cells /cm^2^ in α-MEM (Gibco) containing 10% FCS (Hyclone Laboratories) for 12 days. For CFU-ALP colony formation assays, cells were cultured at a density of <1 × 10^3^ cells /cm^2^ in α-MEM (Gibco) containing 10% FCS (Hyclone Laboratories) with 50 μg/ml ascorbic acid (Sigma-Aldrich) and 10 mM β-glycerophosphate (Sigma-Aldrich) for 12 days. Cell cultures were maintained at 37 °C in a 5% CO_2_ incubator. At the end of the culture period, cells were stained for CFU-F or CFU-ALP activity. (2) For bone nodule formation, cells were cultured at a density of 5 × 10^3^ cells/cm^2^ in α-MEM containing 10% FCS for 7 days, and then cultured in α-MEM containing 10% FCS with 50 μg/ml ascorbic acid and 10 mM β-glycerophosphate for another 14 days. At the end of the culture period, the cells were subjected to von Kossa staining. (3) For the adipogenesis assay, cells were cultured at a density of 1 × 10^3^ cells/cm^2^ in α-MEM containing 10% FCS, 10 nM dexamethasone, 5 μg/ml insulin (Sigma-Aldrich), 100 nM indomethacin (Sigma-Aldrich), and 0.5 mM methylisobutylxanthine (Sigma-Aldrich) for adipocyte induction. Cells were stained with Oil Red O for adipocytes. (4) For the chondrogenesis assay, cells were cultured in 15 ml centrifuge tubes at 1 × 10^3^ cells per tube with StemXVivo Chondrogenic Base Media (catalog number CCM005, R&D Systems) and StemXVivo Chondrogenic Supplement (catalog number CCM006, R&D Systems) for 21 days. At the end of the culture period, a chondrogenic pellet was cryo-sectioned and subjected to Alcian blue staining.

### Histology

The histology study was carried out in the Department of Anatomy at Nanjing Medical University. The alveolar bone including periodontal tissues and teeth were harvested from cadavers that were available in the Department of Anatomy at Nanjing Medical University. As the cadavers had been procured by following a standard ethical protocol, additional ethical clearance was not required to harvest the tissues. The cadavers were embalmed about 6 hours after death. The bodies were kept in a refrigerated chamber before embalming. The diagnosis of periodontitis was relied on the radiographic assessments, including a distance between the cemento-enamel junction (CEJ) and the alveolar bone crest (ABC) exceeding 3 mm. The embalming was done using a 40% formaldehyde solution. The alveolar bone was collected, decalcified in 20% EDTA, embedded in paraffin, and sectioned at 3 μm thickness for three levels (200 μm apart). Sections were stained with H&E for routine histology. The pathologist would confirm the diagnosis with histological sections stained with H & E.

### Immunofluorescence staining

The deparaffinized sections were subjected to antigen retrieval, incubated in 3% hydrogen peroxide for 10 min, blocked in PBS with 10% normal goat serum and 10% Triton X-100 for 1 hour, and then stained overnight at 4 ℃ with mouse anti-LepR (catalog number MAB867, 1:100, R&D Systems) or rabbit anti-CD55 (ab133684, 1:100, Abcam) or mouse anti-collagen I (ab88147, 1:100, Abcam) or rabbit anti-CD358 (ab198034, 1:20, Abcam) or rabbit anti-CXCL12 (97958, 1:200, Cell Signaling Technology) or goat anti-ASPN (ab31303, 1:25, Abcam) or rat anti-CD31 (NB600-1475SS, 1:100, Novus biologicals) or goat anti-ALP (AF2910, 1:100, R & D Systems) or mouse anti-EphrinA1 (sc-377362, 1:200, Santa Cruz) or rabbit anti-Eph A7 (PA1-30296, 1:200, Invitrogen). After rinsing with PBS for 15 minutes, the tissues were incubated at room temperature with goat anti-rabbit Alexa Fluor 488 or goat anti-mouse Alexa Fluor 568 or goat anti-rabbit Alexa Fluor 568 or goat anti-mouse Alexa Fluor 488 or donkey anti-rat Alexa Fluor 405 or goat anti-rabbit Alexa Fluor 647. Slides were mounted with mounting medium containing DAPI (Vector Labs), and images were captured using a Leica DM4000 fluorescence microscope.

### Statistical analysis

No statistical method was used to predetermine sample sizes. Box plots were generated using the R base package and default parameters. Hence, the boxes span the interquartile range (IQR; from the 25th to the 75^th^ percentiles) with the centerline corresponding to the median. The whiskers represent the lowest data point still within 1.5 × IQR of the lower quartile and the highest data point still within 1.5 × IQR of the upper quartile. Violin plots were generated using the beanplot R package, and data distribution bandwidth was estimated by kernel density estimation, as per the built-in 'nrd0' option. One-way analysis of variance with Tukey's multiple comparisons tests were used for multiple group comparisons. Comparisons between the two groups were made using unpaired two-tailed *t*-tests. All statistical analyses and presentations were performed using R.

## Supplementary Material

Supplementary figures.

Supplementary table 1.

Supplementary table 2.

Supplementary table 3.

Supplementary table 4.

Supplementary table 5.

Supplementary table 6.

Supplementary table 7.

Supplementary table 8.

Supplementary table 9.

## References

[1] Pihlstrom BL, Michalowicz BS, Johnson NW (2005). Periodontal diseases. Lancet.

[2] Kassebaum NJ, Bernabe E, Dahiya M, Bhandari B, Murray CJ, Marcenes W (2014). Global burden of severe periodontitis in 1990-2010: a systematic review and meta-regression. J Dent Res.

[3] Tsukasaki M, Takayanagi H (2019). Osteoimmunology: evolving concepts in bone-immune interactions in health and disease. Nat Rev Immunol.

[4] Hajishengallis G (2015). Periodontitis: from microbial immune subversion to systemic inflammation. Nat Rev Immunol.

[5] Dioguardi M, Crincoli V, Laino L, Alovisi M, Sovereto D, Mastrangelo F (2020). The Role of Periodontitis and Periodontal Bacteria in the Onset and Progression of Alzheimer's Disease: A Systematic Review. J Clin Med.

[6] Belkina AC, Azer M, Lee JJ, Elgaali HH, Pihl R, Cleveland M (2020). Single-Cell Analysis of the Periodontal Immune Niche in Type 2 Diabetes. J Dent Res.

[7] Reynolds MA, Kao RT, Camargo PM, Caton JG, Clem DS, Fiorellini JP (2015). Periodontal regeneration - intrabony defects: a consensus report from the AAP Regeneration Workshop. J Periodontol.

[8] Isidor F, Attstrom R, Karring T (1985). Regeneration of alveolar bone following surgical and non-surgical periodontal treatment. J Clin Periodontol.

[9] de Barros FC, Braga FF, Fischer RG, Figueredo CM (2014). Effects of nonsurgical periodontal treatment on the alveolar bone density. Braz Dent J.

[10] Gruber R (2019). Osteoimmunology: Inflammatory osteolysis and regeneration of the alveolar bone. J Clin Periodontol.

[11] Xiao L, Zhou Y, Zhu L, Yang S, Huang R, Shi W (2018). SPHK1-S1PR1-RANKL Axis Regulates the Interactions Between Macrophages and BMSCs in Inflammatory Bone Loss. J Bone Miner Res.

[12] Araujo-Pires AC, Vieira AE, Francisconi CF, Biguetti CC, Glowacki A, Yoshizawa S (2015). IL-4/CCL22/CCR4 axis controls regulatory T-cell migration that suppresses inflammatory bone loss in murine experimental periodontitis. J Bone Miner Res.

[13] Tsukasaki M, Komatsu N, Nagashima K, Nitta T, Pluemsakunthai W, Shukunami C (2018). Host defense against oral microbiota by bone-damaging T cells. Nat Commun.

[14] Mahanonda R, Champaiboon C, Subbalekha K, Sa-Ard-Iam N, Rattanathammatada W, Thawanaphong S (2016). Human Memory B Cells in Healthy Gingiva, Gingivitis, and Periodontitis. J Immunol.

[15] Arron JR, Choi Y (2000). Bone versus immune system. Nature.

[16] Walsh MC, Takegahara N, Kim H, Choi Y (2018). Updating osteoimmunology: regulation of bone cells by innate and adaptive immunity. Nat Rev Rheumatol.

[17] Vegh P, Haniffa M (2018). The impact of single-cell RNA sequencing on understanding the functional organization of the immune system. Brief Funct Genomics.

[18] Blin-Wakkach C, de Vries TJ (2019). Editorial: Advances in Osteoimmunology. Front Immunol.

[19] Condamine T, Dominguez GA, Youn JI, Kossenkov AV, Mony S, Alicea-Torres K (2016). Lectin-type oxidized LDL receptor-1 distinguishes population of human polymorphonuclear myeloid-derived suppressor cells in cancer patients. Sci Immunol.

[20] Guo YE, Li Y, Cai B, He Q, Chen G, Wang M (2021). Phenotyping of immune and endometrial epithelial cells in endometrial carcinomas revealed by single-cell RNA sequencing. Aging (Albany NY).

[21] Tabula Muris C, Overall c, Logistical c, Organ c, processing, Library p (2018). Single-cell transcriptomics of 20 mouse organs creates a Tabula Muris. Nature.

[22] Bian Z, Gong Y, Huang T, Lee CZW, Bian L, Bai Z (2020). Deciphering human macrophage development at single-cell resolution. Nature.

[23] Bondjers C, He L, Takemoto M, Norlin J, Asker N, Hellstrom M (2006). Microarray analysis of blood microvessels from PDGF-B and PDGF-Rbeta mutant mice identifies novel markers for brain pericytes. FASEB J.

[24] Baryawno N, Przybylski D, Kowalczyk MS, Kfoury Y, Severe N, Gustafsson K (2019). A Cellular Taxonomy of the Bone Marrow Stroma in Homeostasis and Leukemia. Cell.

[25] Croft AP, Campos J, Jansen K, Turner JD, Marshall J, Attar M (2019). Distinct fibroblast subsets drive inflammation and damage in arthritis. Nature.

[26] Smith PC, Martinez C, Martinez J, McCulloch CA (2019). Role of Fibroblast Populations in Periodontal Wound Healing and Tissue Remodeling. Front Physiol.

[27] Lambrechts D, Wauters E, Boeckx B, Aibar S, Nittner D, Burton O (2018). Phenotype molding of stromal cells in the lung tumor microenvironment. Nat Med.

[28] Lu EM, Hobbs C, Dyer C, Ghuman M, Hughes FJ (2020). Differential regulation of epithelial growth by gingival and periodontal fibroblasts *in vitro*. J Periodontal Res.

[29] Zhang T, Yu J, Wang G, Zhang R (2021). Amyloid precursor protein binds with TNFRSF21 to induce neural inflammation in Alzheimer's Disease. Eur J Pharm Sci.

[30] Alvarez C, Monasterio G, Cavalla F, Cordova LA, Hernandez M, Heymann D (2019). Osteoimmunology of Oral and Maxillofacial Diseases: Translational Applications Based on Biological Mechanisms. Front Immunol.

[31] Mabuchi Y, Morikawa S, Harada S, Niibe K, Suzuki S, Renault-Mihara F (2013). LNGFR(+)THY-1(+)VCAM-1(hi+) cells reveal functionally distinct subpopulations in mesenchymal stem cells. Stem Cell Reports.

[32] Mebarki M, Iglicki N, Marigny C, Abadie C, Nicolet C, Churlaud G (2021). Development of a human umbilical cord-derived mesenchymal stromal cell-based advanced therapy medicinal product to treat immune and/or inflammatory diseases. Stem Cell Res Ther.

[33] Priglinger E, Strasser J, Buchroithner B, Weber F, Wolbank S, Auer D (2021). Label-free characterization of an extracellular vesicle-based therapeutic. J Extracell Vesicles.

[34] Zhou BO, Yue R, Murphy MM, Peyer JG, Morrison SJ (2014). Leptin-receptor-expressing mesenchymal stromal cells represent the main source of bone formed by adult bone marrow. Cell Stem Cell.

[35] Zhang L, Wei Y, Chi Y, Liu D, Yang S, Han Z (2021). Two-step generation of mesenchymal stem/stromal cells from human pluripotent stem cells with reinforced efficacy upon osteoarthritis rabbits by HA hydrogel. Cell Biosci.

[36] Deng CC, Hu YF, Zhu DH, Cheng Q, Gu JJ, Feng QL (2021). Single-cell RNA-seq reveals fibroblast heterogeneity and increased mesenchymal fibroblasts in human fibrotic skin diseases. Nat Commun.

[37] Supakul S, Yao K, Ochi H, Shimada T, Hashimoto K, Sunamura S (2019). Pericytes as a Source of Osteogenic Cells in Bone Fracture Healing. Int J Mol Sci.

[38] Harrell CR, Simovic Markovic B, Fellabaum C, Arsenijevic A, Djonov V, Volarevic V (2018). Molecular mechanisms underlying therapeutic potential of pericytes. J Biomed Sci.

[39] Xu J, Li D, Hsu CY, Tian Y, Zhang L, Wang Y (2020). Comparison of skeletal and soft tissue pericytes identifies CXCR4(+) bone forming mural cells in human tissues. Bone Res.

[40] Kato K, Dieguez-Hurtado R, Park DY, Hong SP, Kato-Azuma S, Adams S (2018). Pulmonary pericytes regulate lung morphogenesis. Nat Commun.

[41] Zhou Y, Yang D, Yang Q, Lv X, Huang W, Zhou Z (2020). Single-cell RNA landscape of intratumoral heterogeneity and immunosuppressive microenvironment in advanced osteosarcoma. Nat Commun.

[42] Xing X, Yang F, Huang Q, Guo H, Li J, Qiu M (2021). Decoding the multicellular ecosystem of lung adenocarcinoma manifested as pulmonary subsolid nodules by single-cell RNA sequencing. Sci Adv.

[43] Zhang F, Luo K, Rong Z, Wang Z, Luo F, Zhang Z (2017). Periostin Upregulates Wnt/beta-Catenin Signaling to Promote the Osteogenesis of CTLA4-Modified Human Bone Marrow-Mesenchymal Stem Cells. Sci Rep.

[44] Knight MN, Karuppaiah K, Lowe M, Mohanty S, Zondervan RL, Bell S (2018). R-spondin-2 is a Wnt agonist that regulates osteoblast activity and bone mass. Bone Res.

[45] Andersson-Sjoland A, Karlsson JC, Rydell-Tormanen K (2016). ROS-induced endothelial stress contributes to pulmonary fibrosis through pericytes and Wnt signaling. Lab Invest.

[46] Wang N, Deng Y, Liu A, Shen N, Wang W, Du X (2017). Novel Mechanism of the Pericyte-Myofibroblast Transition in Renal Interstitial Fibrosis: Core Fucosylation Regulation. Sci Rep.

[47] Wang YC, Chen Q, Luo JM, Nie J, Meng QH, Shuai W (2019). Notch1 promotes the pericyte-myofibroblast transition in idiopathic pulmonary fibrosis through the PDGFR/ROCK1 signal pathway. Exp Mol Med.

[48] Madel MB, Ibanez L, Wakkach A, de Vries TJ, Teti A, Apparailly F (2019). Immune Function and Diversity of Osteoclasts in Normal and Pathological Conditions. Front Immunol.

[49] Zhang L, Li Z, Skrzypczynska KM, Fang Q, Zhang W, O'Brien SA (2020). Single-Cell Analyses Inform Mechanisms of Myeloid-Targeted Therapies in Colon Cancer. Cell.

[50] Dang T, Liou GY (2018). Macrophage Cytokines Enhance Cell Proliferation of Normal Prostate Epithelial Cells through Activation of ERK and Akt. Sci Rep.

[51] Brulois K, Rajaraman A, Szade A, Nordling S, Bogoslowski A, Dermadi D (2020). A molecular map of murine lymph node blood vascular endothelium at single cell resolution. Nat Commun.

[52] Liu M, Zhang L, Marsboom G, Jambusaria A, Xiong S, Toth PT (2019). Sox17 is required for endothelial regeneration following inflammation-induced vascular injury. Nat Commun.

[53] Marti P, Stein C, Blumer T, Abraham Y, Dill MT, Pikiolek M (2015). YAP promotes proliferation, chemoresistance, and angiogenesis in human cholangiocarcinoma through TEAD transcription factors. Hepatology.

[54] Lopez-Camarillo C, Ocampo EA, Casamichana ML, Perez-Plasencia C, Alvarez-Sanchez E, Marchat LA (2012). Protein kinases and transcription factors activation in response to UV-radiation of skin: implications for carcinogenesis. Int J Mol Sci.

[55] Calicchio R, Buffat C, Mathieu JR, Ben Salem N, Mehats C, Jacques S (2013). Preeclamptic plasma induces transcription modifications involving the AP-1 transcriptional regulator JDP2 in endothelial cells. Am J Pathol.

[56] Jia J, Ye T, Cui P, Hua Q, Zeng H, Zhao D (2016). AP-1 transcription factor mediates VEGF-induced endothelial cell migration and proliferation. Microvasc Res.

[57] Larsson M, Shankar EM, Che KF, Saeidi A, Ellegard R, Barathan M (2013). Molecular signatures of T-cell inhibition in HIV-1 infection. Retrovirology.

[58] Semerad CL, Christopher MJ, Liu F, Short B, Simmons PJ, Winkler I (2005). G-CSF potently inhibits osteoblast activity and CXCL12 mRNA expression in the bone marrow. Blood.

[59] Lorenzo J (2013). Cytokines and the Pathogenesis of Osteoporosis. Osteoporosis.

[60] Marelli-Berg FM, Jarmin SJ (2004). Antigen presentation by the endothelium: a green light for antigen-specific T cell trafficking?. Immunol Lett.

[61] Yang X, Chang Y, Wei W (2016). Endothelial Dysfunction and Inflammation: Immunity in Rheumatoid Arthritis. Mediators Inflamm.

[62] Smyth LCD, Rustenhoven J, Park TI, Schweder P, Jansson D, Heppner PA (2018). Unique and shared inflammatory profiles of human brain endothelia and pericytes. J Neuroinflammation.

[63] Chen WY, Wu YH, Tsai TH, Li RF, Lai AC, Li LC (2021). Group 2 innate lymphoid cells contribute to IL-33-mediated alleviation of cardiac fibrosis. Theranostics.

[64] Caetano AJ, Yianni V, Volponi A, Booth V, D'Agostino EM, Sharpe P (2021). Defining human mesenchymal and epithelial heterogeneity in response to oral inflammatory disease. Elife.

[65] Zhang L, Yu X, Zheng L, Zhang Y, Li Y, Fang Q (2018). Lineage tracking reveals dynamic relationships of T cells in colorectal cancer. Nature.

[66] Williams DW, Greenwell-Wild T, Brenchley L, Dutzan N, Overmiller A, Sawaya AP (2021). Human oral mucosa cell atlas reveals a stromal-neutrophil axis regulating tissue immunity. Cell.

[67] Shelby A, Pendleton C, Thayer E, Johnson GK, Xie XJ, Brogden KA (2020). PD-L1 correlates with chemokines and cytokines in gingival crevicular fluid from healthy and diseased sites in subjects with periodontitis. BMC Res Notes.

[68] Dyck L, Mills KHG (2017). Immune checkpoints and their inhibition in cancer and infectious diseases. Eur J Immunol.

[69] He F, Zhou Y, Wang X, Li L, Geng Y, Wang Z (2018). Functional Polymorphisms of CTLA4 Associated with Aggressive Periodontitis in the Chinese Han Population. Cell Physiol Biochem.

[70] Zhang J, Wang CM, Zhang P, Wang X, Chen J, Yang J (2016). Expression of programmed death 1 ligand 1 on periodontal tissue cells as a possible protective feedback mechanism against periodontal tissue destruction. Mol Med Rep.

[71] Zhu S, Bennett S, Kuek V, Xiang C, Xu H, Rosen V (2020). Endothelial cells produce angiocrine factors to regulate bone and cartilage via versatile mechanisms. Theranostics.

[72] Tompkins KA (2016). The osteoimmunology of alveolar bone loss. Connect Tissue Res.

[73] Humbert P, Brennan MA, Davison N, Rosset P, Trichet V, Blanchard F (2019). Immune Modulation by Transplanted Calcium Phosphate Biomaterials and Human Mesenchymal Stromal Cells in Bone Regeneration. Front Immunol.

[74] Zhang F, Wei K, Slowikowski K, Fonseka CY, Rao DA, Kelly S (2019). Defining inflammatory cell states in rheumatoid arthritis joint synovial tissues by integrating single-cell transcriptomics and mass cytometry. Nat Immunol.

[75] Boland BS, He Z, Tsai MS, Olvera JG, Omilusik KD, Duong HG (2020). Heterogeneity and clonal relationships of adaptive immune cells in ulcerative colitis revealed by single-cell analyses. Sci Immunol.

[76] Zhong L, Yao L, Tower RJ, Wei Y, Miao Z, Park J (2020). Single cell transcriptomics identifies a unique adipose lineage cell population that regulates bone marrow environment. Elife.

[77] McDonald MM, Khoo WH, Ng PY, Xiao Y, Zamerli J, Thatcher P (2021). Osteoclasts recycle via osteomorphs during RANKL-stimulated bone resorption. Cell.

[78] Jones S, Horwood N, Cope A, Dazzi F (2007). The antiproliferative effect of mesenchymal stem cells is a fundamental property shared by all stromal cells. J Immunol.

[79] Li Y, Lin F (2012). Mesenchymal stem cells are injured by complement after their contact with serum. Blood.

[80] Gavin C, Meinke S, Heldring N, Heck KA, Achour A, Iacobaeus E (2019). The Complement System Is Essential for the Phagocytosis of Mesenchymal Stromal Cells by Monocytes. Front Immunol.

[81] Weng L, Hu X, Kumar B, Garcia M, Todorov I, Jung X (2016). Identification of a CD133-CD55- population functions as a fetal common skeletal progenitor. Sci Rep.

[82] Sculean A, Chapple IL, Giannobile WV (2015). Wound models for periodontal and bone regeneration: the role of biologic research. Periodontol 2000.

[83] Della Coletta BB, Jacob TB, Moreira LAC, Pomini KT, Buchaim DV, Eleuterio RG (2021). Photobiomodulation Therapy on the Guided Bone Regeneration Process in Defects Filled by Biphasic Calcium Phosphate Associated with Fibrin Biopolymer. Molecules.

[84] Lei G, Wang Y, Yu Y, Li Z, Lu J, Ge X (2020). Dentin-Derived Inorganic Minerals Promote the Osteogenesis of Bone Marrow-Derived Mesenchymal Stem Cells: Potential Applications for Bone Regeneration. Stem Cells Int.

[85] Nwadozi E, Rudnicki M, Haas TL (2020). Metabolic Coordination of Pericyte Phenotypes: Therapeutic Implications. Front Cell Dev Biol.

[86] Tonna S, Sims NA (2014). Talking among ourselves: paracrine control of bone formation within the osteoblast lineage. Calcif Tissue Int.

[87] Matsuo K, Otaki N (2012). Bone cell interactions through Eph/ephrin: bone modeling, remodeling and associated diseases. Cell Adh Migr.

[88] Lindsey RC, Rundle CH, Mohan S (2018). Role of IGF1 and EFN-EPH signaling in skeletal metabolism. J Mol Endocrinol.

[89] Mimche PN, Lee CM, Mimche SM, Thapa M, Grakoui A, Henkemeyer M (2018). EphB2 receptor tyrosine kinase promotes hepatic fibrogenesis in mice via activation of hepatic stellate cells. Sci Rep.

[90] Lagares D, Ghassemi-Kakroodi P, Tremblay C, Santos A, Probst CK, Franklin A (2017). ADAM10-mediated ephrin-B2 shedding promotes myofibroblast activation and organ fibrosis. Nat Med.

[91] Giaginis C, Tsoukalas N, Bournakis E, Alexandrou P, Kavantzas N, Patsouris E (2014). Ephrin (Eph) receptor A1, A4, A5 and A7 expression in human non-small cell lung carcinoma: associations with clinicopathological parameters, tumor proliferative capacity and patients' survival. BMC Clin Pathol.

[92] Gajdzis M, Theocharis S, Gajdzis P, Cassoux N, Gardrat S, Donizy P (2020). Ephrin Receptors (Eph): EphA1, EphA5, and EphA7 Expression in Uveal Melanoma-Associations with Clinical Parameters and Patient Survival. Life (Basel).

[93] Turner RT, Iwaniec UT, Marley K, Sibonga JD (2010). The role of mast cells in parathyroid bone disease. J Bone Miner Res.

[94] Lotinun S, Sibonga JD, Turner RT (2005). Evidence that the cells responsible for marrow fibrosis in a rat model for hyperparathyroidism are preosteoblasts. Endocrinology.

[95] Kisseleva T, Brenner DA (2008). Mechanisms of fibrogenesis. Exp Biol Med (Maywood).

[96] Ducy P, Schinke T, Karsenty G (2000). The osteoblast: a sophisticated fibroblast under central surveillance. Science.

[97] He X-T, Wu R-X, Chen F-M (2020). Periodontal tissue engineering and regeneration. Principles of Tissue Engineering.

[98] Coelho NM, McCulloch CA (2016). Contribution of collagen adhesion receptors to tissue fibrosis. Cell Tissue Res.

[99] Liu L, Guo S, Shi W, Liu Q, Huo F, Wu Y (2020). Bone Marrow Mesenchymal Stem Cell-Derived Small Extracellular Vesicles Promote Periodontal Regeneration. Tissue Eng Part A.

[100] Saha N, Robev D, Mason EO, Himanen JP, Nikolov DB (2018). Therapeutic potential of targeting the Eph/ephrin signaling complex. Int J Biochem Cell Biol.

[101] Zahr AA, Salama ME, Carreau N, Tremblay D, Verstovsek S, Mesa R (2016). Bone marrow fibrosis in myelofibrosis: pathogenesis, prognosis and targeted strategies. Haematologica.

[102] Joshi N, Watanabe S, Verma R, Jablonski RP, Chen CI, Cheresh P (2020). A spatially restricted fibrotic niche in pulmonary fibrosis is sustained by M-CSF/M-CSFR signalling in monocyte-derived alveolar macrophages. Eur Respir J.

[103] Arthur A, Gronthos S (2021). Eph-Ephrin Signaling Mediates Cross-Talk Within the Bone Microenvironment. Front Cell Dev Biol.

[105] Maiuolo J, Muscoli C, Gliozzi M, Musolino V, Carresi C, Paone S (2021). Endothelial Dysfunction and Extra-Articular Neurological Manifestations in Rheumatoid Arthritis. Biomolecules.

[106] Wang H, Chen Y, Li W, Sun L, Chen H, Yang Q (2020). Effect of VEGFC on lymph flow and inflammation-induced alveolar bone loss. J Pathol.

[107] Danese S, Dejana E, Fiocchi C (2007). Immune regulation by microvascular endothelial cells: directing innate and adaptive immunity, coagulation, and inflammation. J Immunol.

[108] Mai J, Virtue A, Shen J, Wang H, Yang XF (2013). An evolving new paradigm: endothelial cells-conditional innate immune cells. J Hematol Oncol.

[109] Shao Y, Saredy J, Yang WY, Sun Y, Lu Y, Saaoud F (2020). Vascular Endothelial Cells and Innate Immunity. Arterioscler Thromb Vasc Biol.

[110] Li Q, Zhu Z, Wang L, Lin Y, Fang H, Lei J (2021). Single-cell transcriptome profiling reveals vascular endothelial cell heterogeneity in human skin. Theranostics.

[111] Cavalla F, Letra A, Silva RM, Garlet GP (2021). Determinants of Periodontal/Periapical Lesion Stability and Progression. J Dent Res.

[112] Adam S, Simon N, Steffen U, Andes FT, Scholtysek C, Muller DIH (2020). JAK inhibition increases bone mass in steady-state conditions and ameliorates pathological bone loss by stimulating osteoblast function. Sci Transl Med.

[113] Moreno JL, Kaczmarek M, Keegan AD, Tondravi M (2003). IL-4 suppresses osteoclast development and mature osteoclast function by a STAT6-dependent mechanism: irreversible inhibition of the differentiation program activated by RANKL. Blood.

[114] Qian SJ, Huang QR, Chen RY, Mo JJ, Zhou LY, Zhao Y (2021). Single-Cell RNA Sequencing Identifies New Inflammation-Promoting Cell Subsets in Asian Patients With Chronic Periodontitis. Front Immunol.

[115] Huang N, Perez P, Kato T, Mikami Y, Okuda K, Gilmore RC (2021). SARS-CoV-2 infection of the oral cavity and saliva. Nat Med.

[116] Hedin CA, Larsson A (1984). The ultrastructure of the gingival epithelium in smokers' melanosis. J Periodontal Res.

[117] Feller L, Masilana A, Khammissa RA, Altini M, Jadwat Y, Lemmer J (2014). Melanin: the biophysiology of oral melanocytes and physiological oral pigmentation. Head Face Med.

[118] Righi A, Betts CM, Marchetti C, Marucci G, Montebugnoli L, Prati C (2006). Merkel cells in the oral mucosa. Int J Surg Pathol.

[119] Bonnet JJ, Costentin J (1989). Correlation between (3H)dopamine specific uptake and (3H)GBR 12783 specific binding during the maturation of rat striatum. Life Sci.

[120] Wang H, Hu Z, Wu J, Mei Y, Zhang Q, Zhang H (2019). Sirt1 Promotes Osteogenic Differentiation and Increases Alveolar Bone Mass via Bmi1 Activation in Mice. J Bone Miner Res.

[121] Chen S, Zhou Y, Chen Y, Gu J (2018). fastp: an ultra-fast all-in-one FASTQ preprocessor. Bioinformatics.

[122] Smith T, Heger A, Sudbery I (2017). UMI-tools: modeling sequencing errors in Unique Molecular Identifiers to improve quantification accuracy. Genome Res.

[123] Dobin A, Davis CA, Schlesinger F, Drenkow J, Zaleski C, Jha S (2013). STAR: ultrafast universal RNA-seq aligner. Bioinformatics.

[124] Korsunsky I, Millard N, Fan J, Slowikowski K, Zhang F, Wei K (2019). Fast, sensitive and accurate integration of single-cell data with Harmony. Nat Methods.

[125] Vento-Tormo R, Efremova M, Botting RA, Turco MY, Vento-Tormo M, Meyer KB (2018). Single-cell reconstruction of the early maternal-fetal interface in humans. Nature.

[126] Aibar S, Gonzalez-Blas CB, Moerman T, Huynh-Thu VA, Imrichova H, Hulselmans G (2017). SCENIC: single-cell regulatory network inference and clustering. Nat Methods.

[127] Yaari G, Bolen CR, Thakar J, Kleinstein SH (2013). Quantitative set analysis for gene expression: a method to quantify gene set differential expression including gene-gene correlations. Nucleic Acids Res.

[128] Cao J, Spielmann M, Qiu X, Huang X, Ibrahim DM, Hill AJ (2019). The single-cell transcriptional landscape of mammalian organogenesis. Nature.

