# Supplementary material for: Single-cell RNA landscape of the osteoimmunology microenvironment in periodontitis
Source: Theranostics. 2022 Jan 1;12(3):1074–96. doi: 10.7150/thno.65694 (PMC8771561; doi:10.7150/thno.65694)
Supplement: Supplementary file 1 — Supplementary figures. [file thnov12p1074s1.pdf]

Figure S1

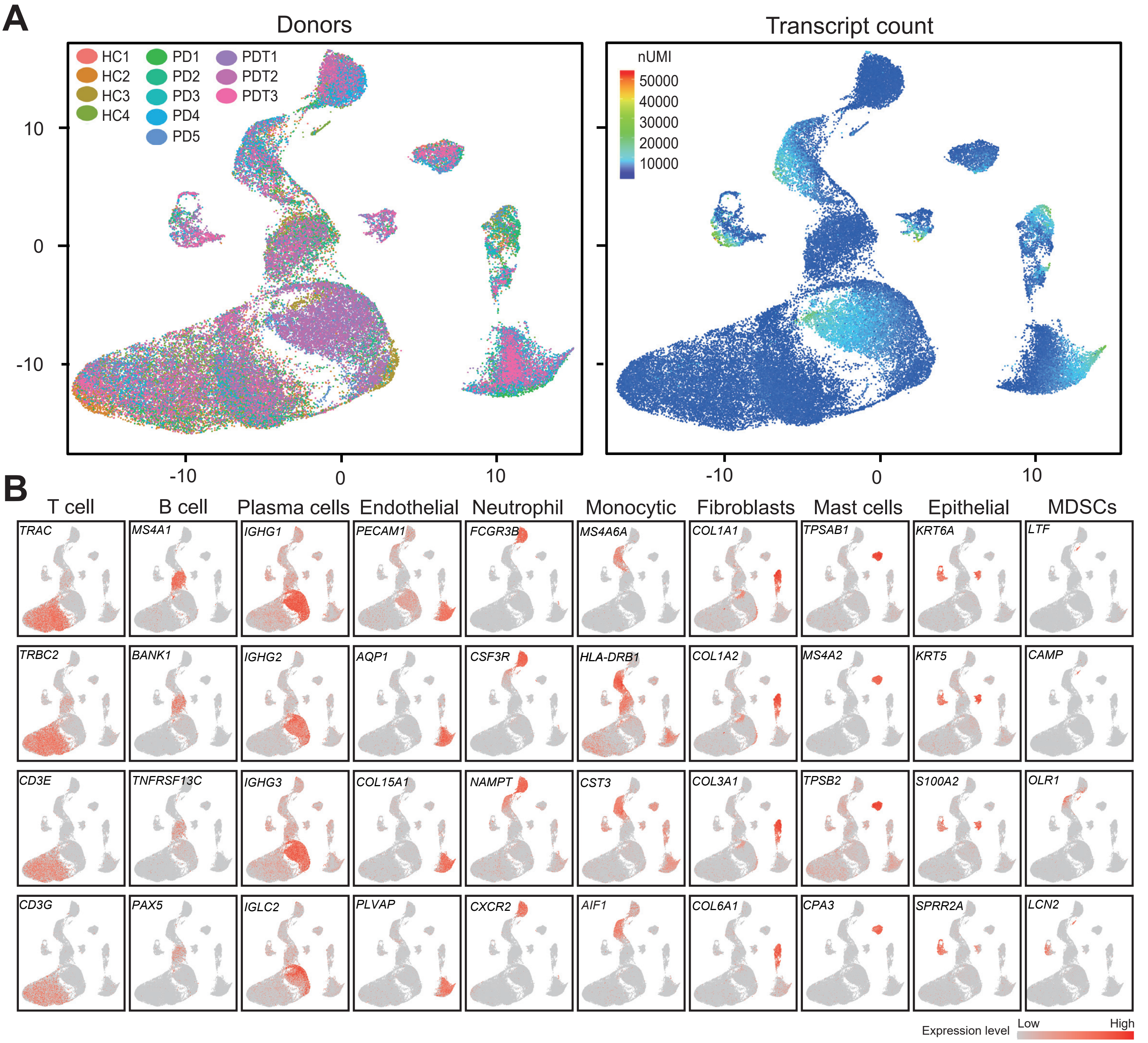

Figure S1. The overview of clustering results related to Figure 1

(A) UMAP of the 51248 cells, annotated and colored by (left to right): the corresponding donors (HCs, PDs, and PDTs) and the number of transcripts (UMIs) detected in that cell (log scale as defined in the inset). MDSCs: Myeloid-derived suppressor cells.

(B) UMAP plots pseudocolored for expression of the marker genes from Fig. 1C (top row) and of 3 additional marker genes (3 bottom rows) for the major cell lineages. Show are, as markers for T cells: genes encoding T-Cell Receptor Alpha Constant (TRAC), T Cell Receptor Beta Constant 2 (TRBC2), CD3e Molecule (CD3E) and CD3g Molecule (CD3G); for B cells: genes encoding Membrane Spanning 4-Domains A1 (MS4A1), B cell scaffold protein with ankyrin repeats 1 (BANK1), TNF Receptor Superfamily Member 13C (TNFRSF13C), Paired Box 5 (PAX5); for Plasma cells: genes encoding Immunoglobulin Heavy Constant Gamma 1 (IGHG1), Immunoglobulin Heavy Constant Gamma 2 (IGHG2), Immunoglobulin Heavy Constant Gamma 3 (IGHG3) and Immunoglobulin Lambda Constant 2 (IGLC2); for endothelial cells: genes encoding Platelet And Endothelial Cell Adhesion Molecule 1 (PECAM1), Aquaporin 1 (AQP1), Collagen type XV alpha 1 chain (COL15A1) and Plasmalemma Vesicle Associated Protein (PLVAP); for neutrophils: genes encoding Fc fragment of IgG receptor IIIb (FCGR3B), Colony stimulating factor 3 receptor (CSF3R), Nicotinamide Phosphoribosyltransferase (NAMPT) and C-X-C Motif Chemokine Receptor 2 (CXCR2); for monocytic cells: genes encoding Membrane Spanning 4-Domains A6A (MS4A6A), Major Histocompatibility Complex, Class II, DR Beta 1 (HLA-DRB1), Cystatin C (CST3) and Allograft Inflammatory Factor 1 (AIF1); for fibroblasts: genes encoding Collagen type I alpha 1 (COL1A1), Collagen type I alpha 2 (COL1A2), Collagen type III alpha 1 (COL3A1) and Collagen type VI alpha 1 (COL6A1); for mast cells: genes encoding Tryptase alpha/beta 1 (TPSAB1), Membrane spanning 4-domains A2 (MS4A2), Tryptase beta 2 (TPSB2) and Carboxypeptidase A3 (CPA3); for Epithelial cells: genes encoding Keratin 6A (KRT6A), keratin 5 (KRT5), S100 calcium binding protein A2 (S100A2) and Small proline rich protein 2A (SPRR2A); for MDSCs: genes encoding Lactotransferrin (LTF), Cathelicidin antimicrobial peptide (CAMP), oxidized low density lipoprotein receptor 1 (OLR1) and Lipocalin 2 (LCN2).

Figure S2

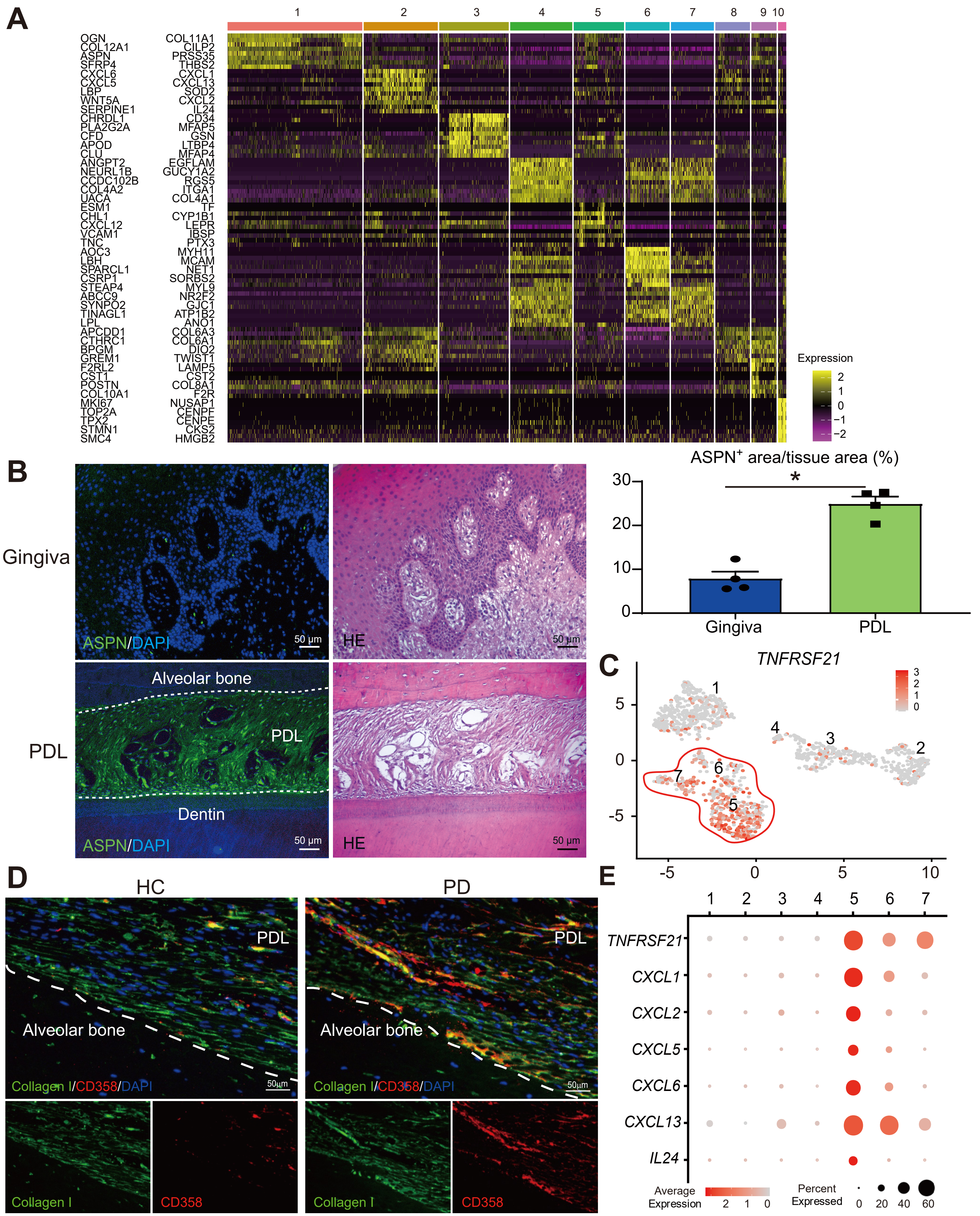

Figure S2. Characterization of Fibroblasts related to Figure 2

(A) Heatmap of the relative expression level of the top 8-10 differentially expressed maker genes identified in the fibroblast clusters in Figure 2A. **(B)** Representative images of immunofluorescence staining of gingiva (top left panels) and PDL (bottom left panels) from HC sample for showing the location of ASPN (green). Scale bar = 50 µm. Adjacent paraffin sections were stained with H & E staining to show the location. The top right panel shows quantification of ASPN^{+} area/tissue area (%) in gingiva and PDL.

(C) UMAP as in Figure 2B colored by expression of TNFRSF21. Red contour: $TNFRSF21^{+}$ fibroblasts (subcluster 4-6).

(D) Representative images of immunofluorescence staining of periodontal tissues from HC (left panels) and PD samples (right panels) for double fluorescent analysis of Collagen I (green) and CD358 (red) expression. Top panel: Merged; bottom left: Collagen I; bottom right: CD358. Scale bar = 50μm.

(E) Bubble plot showing expressions (dots) of the selected marker genes (columns) in each subcluster (rows, as in Figure 2B). Dot colored by the average expression level, and dot size proportional to the percentage expression.

Figure S3

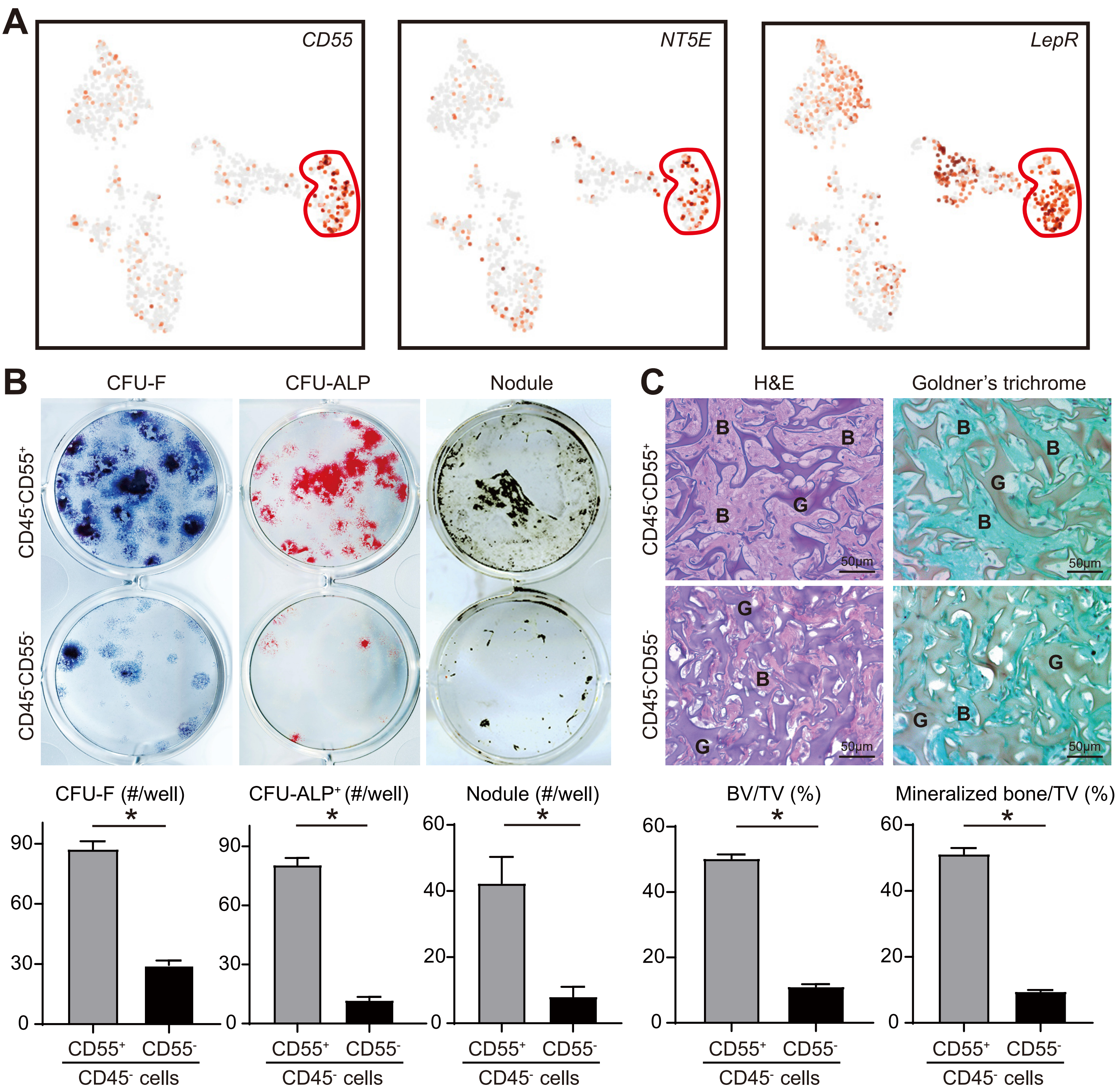

Figure S3. Characterization of CD55^{+} MSCs in periodontal tissue

(A) UMAP as in Figure 2B colored by expression of *CD55*, *NT5E*, and *LepR* (from left to right). Red contour: CD55^{+} MSC (subcluster 2).

(B) Representative images of in vitro assays to determine OB differentiation potential of CD45^{-}CD55^{+} cells compared with those of CD45^{-}CD55^{-} cells. Periodontal cells of a clinically healthy donor were collected and stained with various antibodies and subjected to flow cytometry sorting. CD45^{-}CD55^{+} cells and CD45^{-}CD55^{-} cells were collected and subjected to the following assays. For osteogenic induction, colony-forming units (CFU-F, left)/alkaline phosphatase (ALP)-positive colony-forming units (CFU-ALP, middle)/nodules (right) formation were examined.

(C) CD45^{-}CD55^{+} cells and CD45^{-}CD55^{-} cells were transplanted by subcutaneous surgical implantation into recipient SCID mice. 4 weeks later, the implants were harvested. Upper panels: Representative images of H&E staining (left) and Goldner's Trichrome staining (right) of serial sections of paraffin-embedded ectopic bones. B: bone. G: GelFoam. Bar = 50 μm. Bottom panels: Histomorphometric analysis of bone volume to tissue volume (%) in H&E-stained sections and mineralized bone to tissue area (%) in Goldner's Trichrome staining .

Figure S4

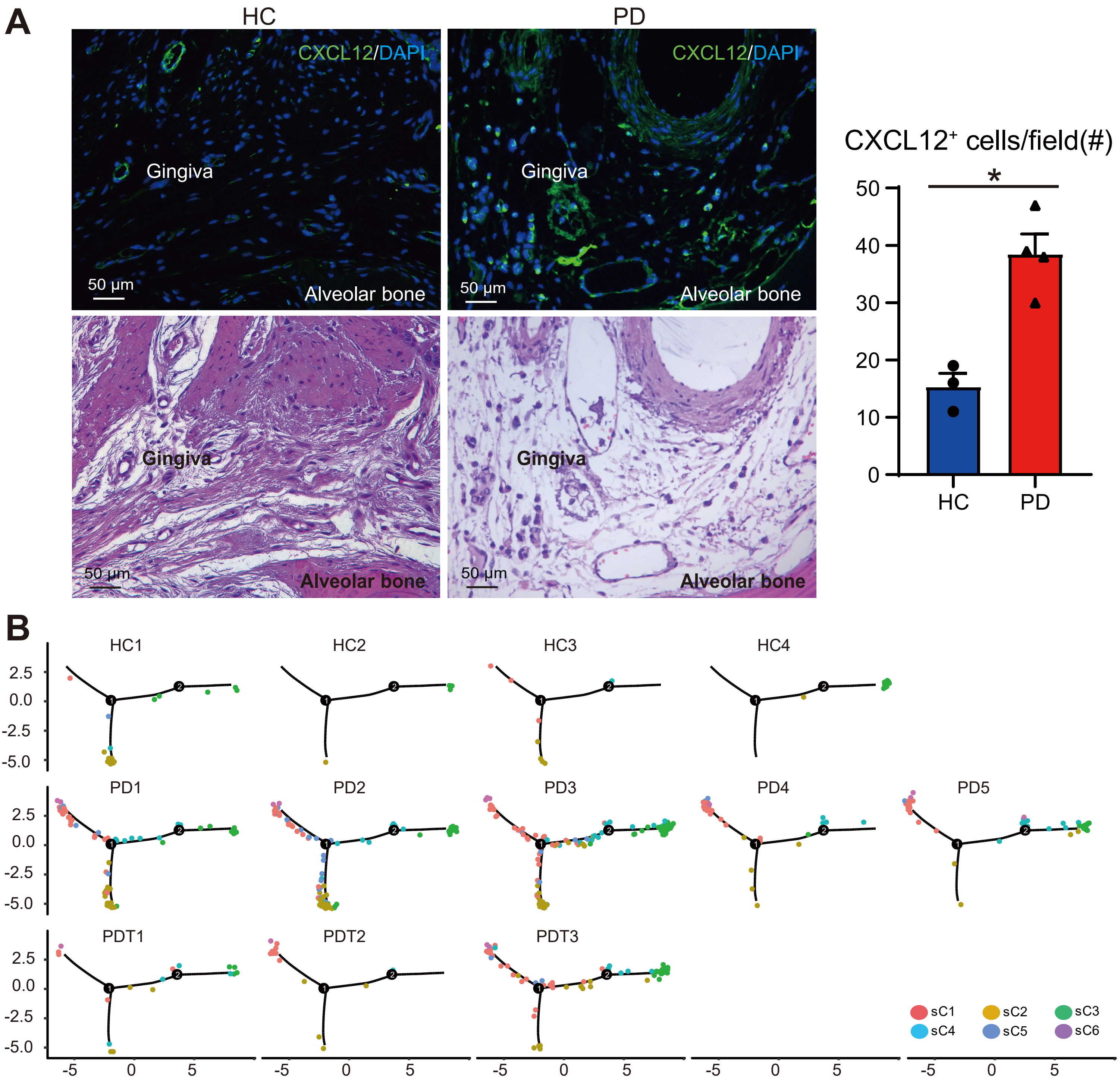

Figure S4. CXCL12 expression in periodontal tissue and the pseudotime trajectory plots from distinct donors related to Figure 3

(A) Representative images of immunofluorescence staining of periodontal tissues from HC (left panel) and PD samples (right panel) for fluorescent analysis of CXCL12 (green). Adjacent paraffin sections were stained with H & E staining to show the location. Scale bar = 50 μm. The right panel shows the quantification of CXCL12^{+} cells.

(B) The distribution of single cell colored according to subclusters as in Figure 3A from the corresponding donors (HCs, PDs, and PDTs) mapped onto pseudotime trajectory plots as in Figure 3C.

Figure S5

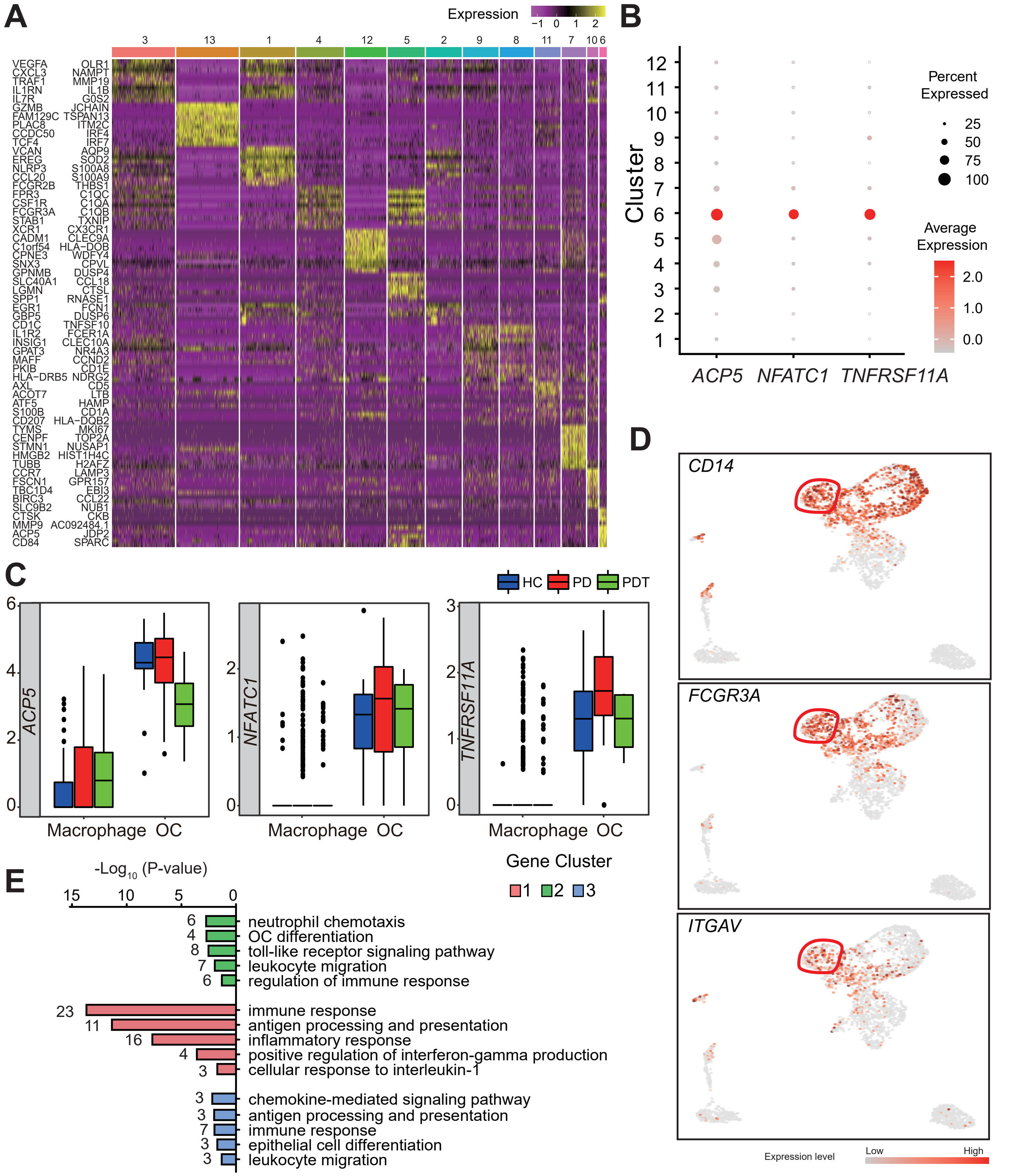

Figure S5. Definition of monocytic clusters and characterization of Macrophages and OCs related to Figure 4

(A) Heatmap of the relative expression level of the top 8-10 differentially expressed maker genes identified in the monocytic clusters in Figure 4A.

(B) Bubble plot revealing three representative genes expression of OCs (cluster 6). Dot colored by the average expression level, and dot size proportional to the percentage expression. OCs: osteoclasts.

(C) Index box plots comparing average expression of three representative genes in macrophages and OCs from HC (blue), PD (red), and PDT (green) samples. The plot center, box, whiskers, and points corresponding to the median, IQR, 1.5 × IQR and >1.5× IQR, respectively. OCs: osteoclasts.

(D) UMAP as in Figure 4A colored by expression of CD14, FCGR3A, and ITGAV (from top to bottom). Red contour: Macrophages-cluster 5.

(E) Pathways enrichment analysis associated with genes clusters in Figure 4E

Figure S6

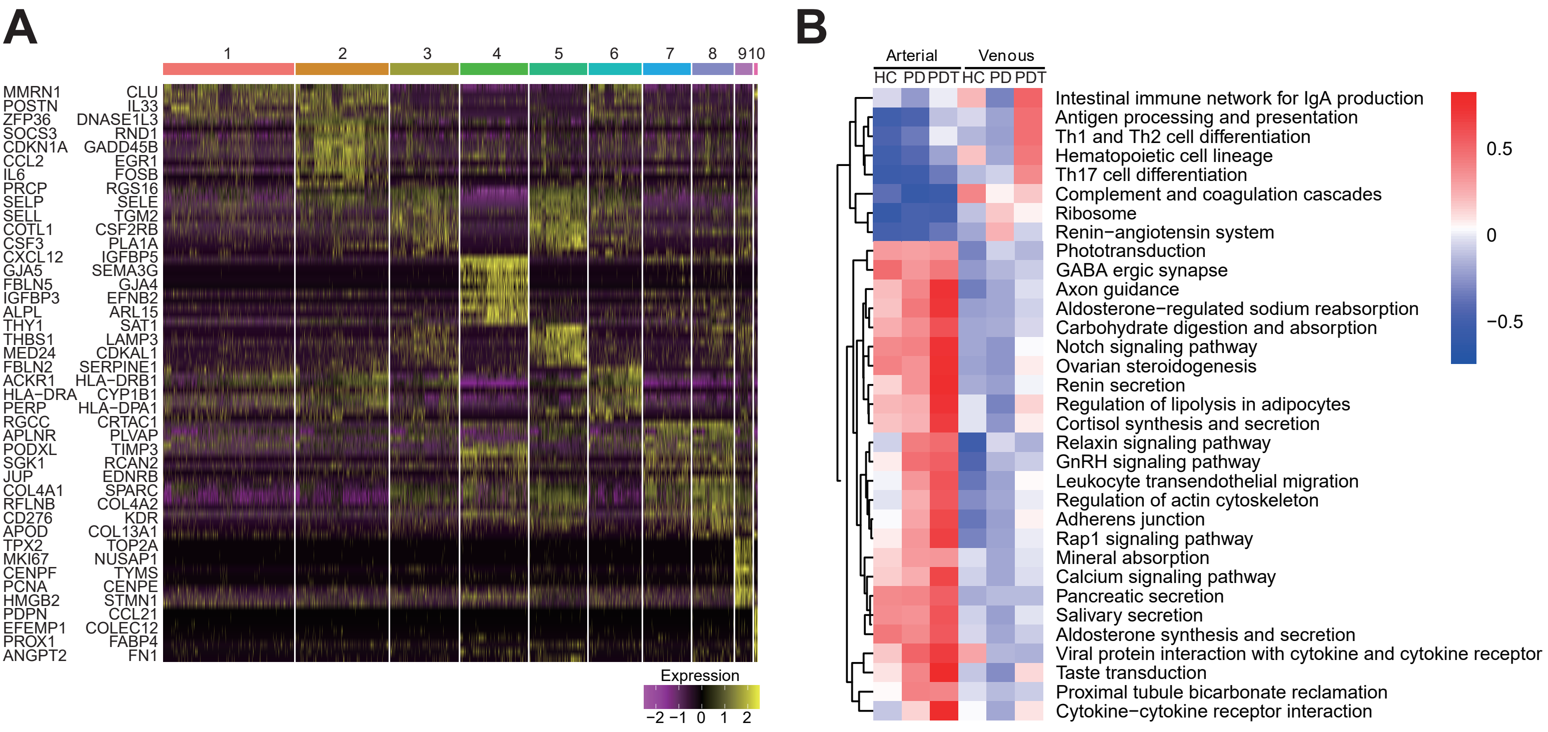

Figure S6. Characterization of ECs related to Figure 5

(A) Heatmap of the relative expression level of the top 8-10 differentially expressed maker genes identified in the endothelial clusters in Figure 5A.

(B) Heatmap of the QuSAGE activity for non-metabolism-related pathways that are associated with differentially expressed genes between the Arterial and Venous ECs clusters from HC, PD, and PDT samples (labels on the top). Red indicating increased average expression of genes in pathways. EC: Endothelial Cell.

Figure S7

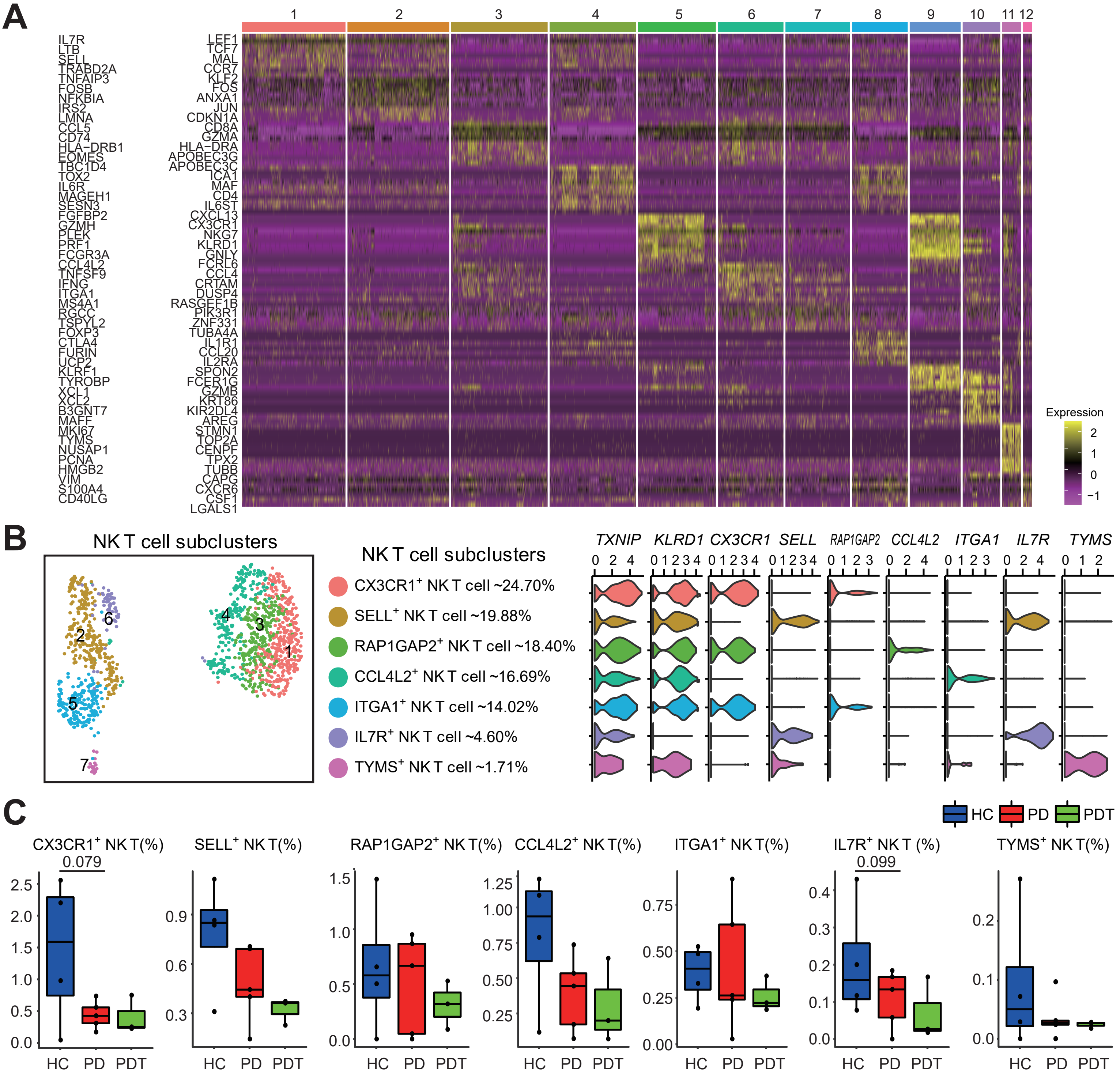

Figure S7. The heterogeneity of T cells and NK T cells

(A) Heatmap of the relative expression level of the top 8-10 differentially expressed maker genes identified in the T cell clusters in Figure 6A.

(B) Left panel: UMAP of 1348 NK T cells, annotated and colored by clustering with the label of each subcluster. Center panel: identified subclusters of NK T cells with the percentages shown. Right panel: Violin plots showing distinct expressions of the selected marker genes (row) in each subcluster of NK T cells as in the center panel.

(C) The box plots showing the percentage of cells for each of seven subclusters of NK T cells as in (A) from HC (blue, n=610), PD (red, n=479), and PDT (green, n=259) samples with plot center, box, whiskers, and points corresponding to median, IQR, 1.5 × IQR and >1.5× IQR, respectively. Data were analyzed using t test (two-tailed, two sample, equal variance)

Figure S8

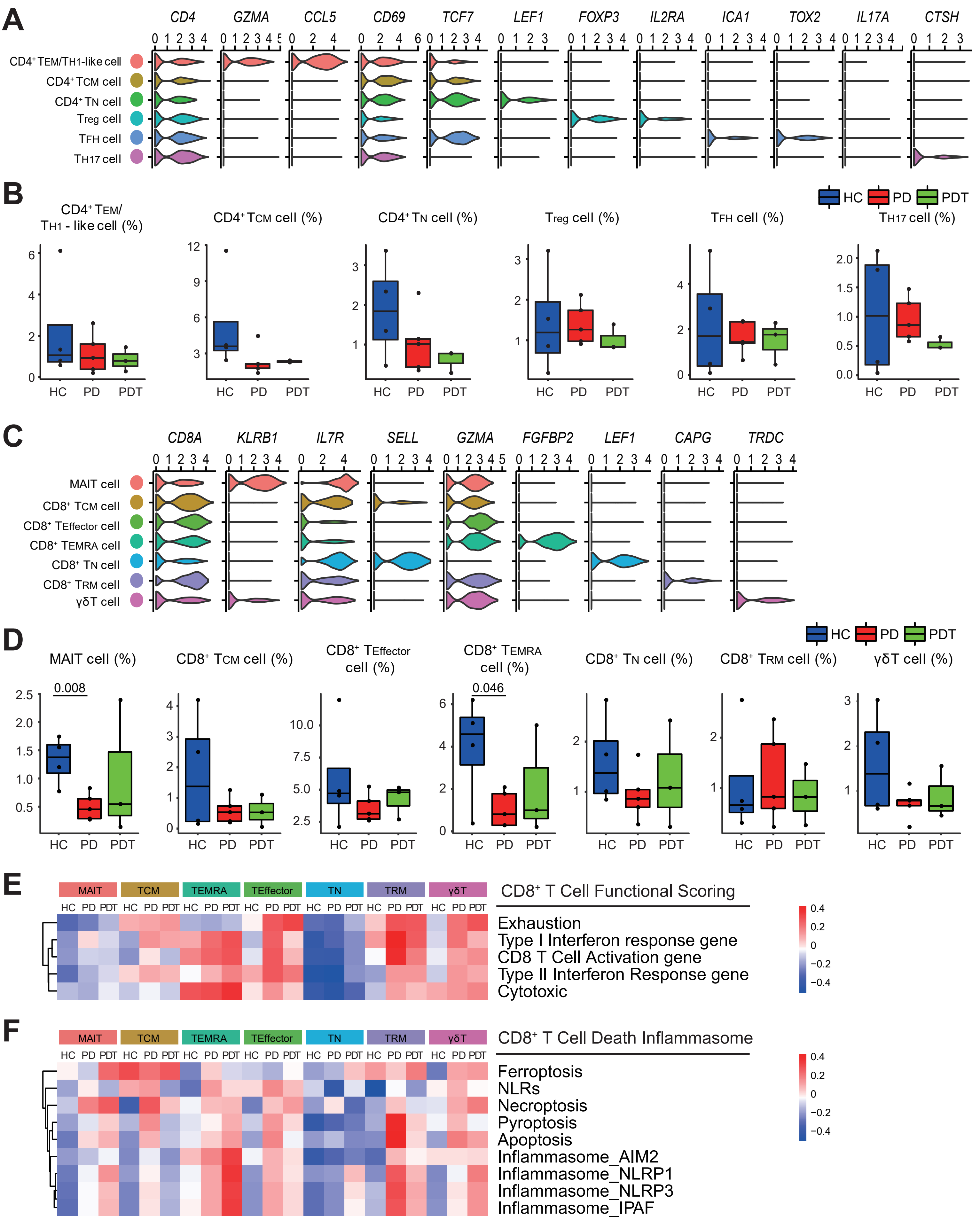
A
CD4
GZMA
CCL5
CD69
TCF7
LEF1
FOXP3
IL2RA
ICA1
TOX2
IL17A
CTSH
CD4+ TEM/TH1-like cell
CD4+ TCM cell
CD4+ TN cell
Treg cell
TFH cell
TH17 cell
B
HC
PD
PDT
CD4+ TEM/ TH1 - like cell (%)
CD4+ TCM cell (%)
CD4+ TN cell (%)
Treg cell (%)
TFH cell (%)
TH17 cell (%)
C
CD8A
KLRB1
IL7R
SELL
GZMA
FGFBP2
LEF1
CAPG
TRDC
MAIT cell
CD8+ TCM cell
CD8+ TEffector cell
CD8+ TEMRA cell
CD8+ TN cell
CD8+ TRM cell
γδT cell
D
MAIT cell (%)
0.008
CD8+ TCM cell (%)
CD8+ TEffector cell (%)
CD8+ TEMRA cell (%)
0.046
CD8+ TN cell (%)
CD8+ TRM cell (%)
γδT cell (%)
E
MAIT
TCM
TEMRA
TEffector
TN
TRM
γδT
CD8+ T Cell Functional Scoring
Exhaustion
Type I Interferon response gene
CD8 T Cell Activation gene
Type II Interferon Response gene
Cytotoxic
F
CD8+ T Cell Death Inflammasome
Ferroptosis
NLRs
Necroptosis
Pyroptosis
Apoptosis
Inflammasome_AIM2
Inflammasome_NLRP1
Inflammasome_NLRP3
Inflammasome_IPAF

Figure S8. The heterogeneity of $CD4^+$ and $CD8^+$ T cell, and the characterization of $CD8^+$ T cell

(A) Violin plots showing distinct expressions of the selected marker genes (row) in each subcluster (labels on left) of $CD4^+$ T cells as in Figure 6C.

(B) The box plots showing the percentage of cells for each subcluster of $CD4^+$ T cells from HC (blue, n=2266), PD (red, n=1637), and PDT (green, n=1163) samples with plot center, box, whiskers, and points corresponding to the median, IQR, 1.5 × IQR and >1.5× IQR, respectively. Data were analyzed using t test (two-tailed, two sample, equal variance).

(C) Violin plots showing distinct expressions of the selected marker genes (row) in each subcluster (labels on left) of $CD8^+$ T cells as in Figure 6D.

(D) The box plots showing the percentage of cells for each subcluster of $CD8^+$ T cells from HC (blue, n=2604), PD (red, n=1695), and PDT (green, n=1652) samples with plot center, box, whiskers, and points corresponding to the median, IQR, 1.5 × IQR and >1.5× IQR, respectively. Data were analyzed using t test (two-tailed, two sample, equal variance).

(E) Heatmap of the QuSAGE activity for the gene modules (labels on the right) that associated with cell functional genes among seven subclusters of $CD8^+$ T cells from HC, PD, and PDT samples (labels on the top). Red indicates increased average expression of genes in the modules.

(F) Heatmap of the QuSAGE activity for gene modules (labels on the right) that associated with cell death inflammasome genes among seven subclusters of $CD8^+$ T cells from HC, PD, and PDT samples (labels on the top). Red indicates an increased average expression of genes in the modules.

Figure S9

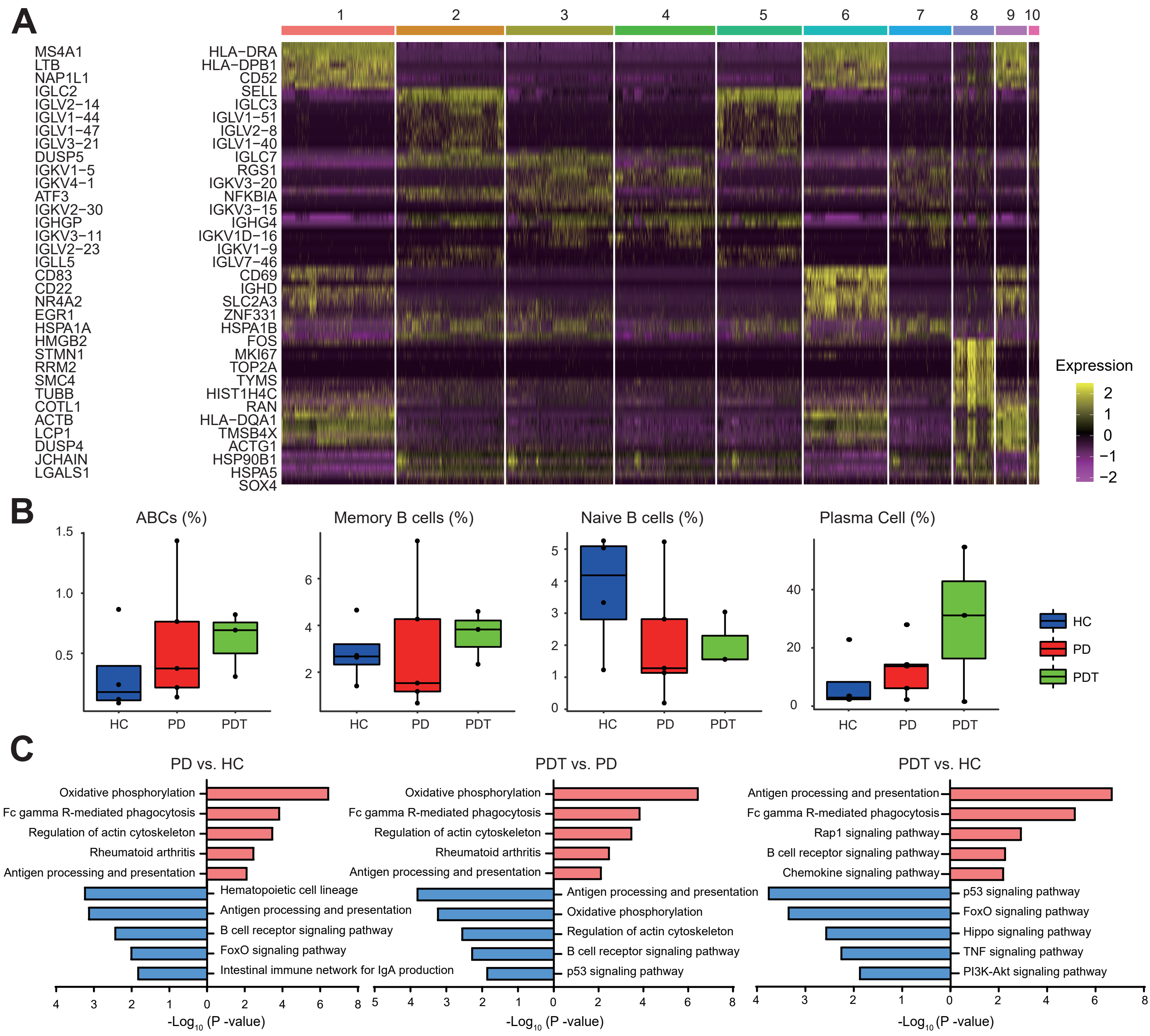

Figure S9. Characterization of B & Plasma cells related to Figure 6

(A) Heatmap of the relative expression level of the top 8-10 differentially expressed maker genes identified in the B & Plasma cells clusters in Figure 6G.

(B) The box plots showing the percentage of cells for each of four subclusters of B & Plasma cells, as in Figure 6G to I, from (blue, n=1039), PD (red, n=1078), and PDT (green, n=1131) samples with plot center, box, whiskers, and points corresponding to the median, IQR, 1.5 × IQR and >1.5× IQR, respectively. Data were analyzed using t test (two-tailed, two sample, equal variance).

(C) Pathways enrichment analysis associated with genes upregulated (red) and downregulated (blue) genes in （from left to right）PD vs. HC, PDT vs. PD and PDT vs. HC.

Figure S10

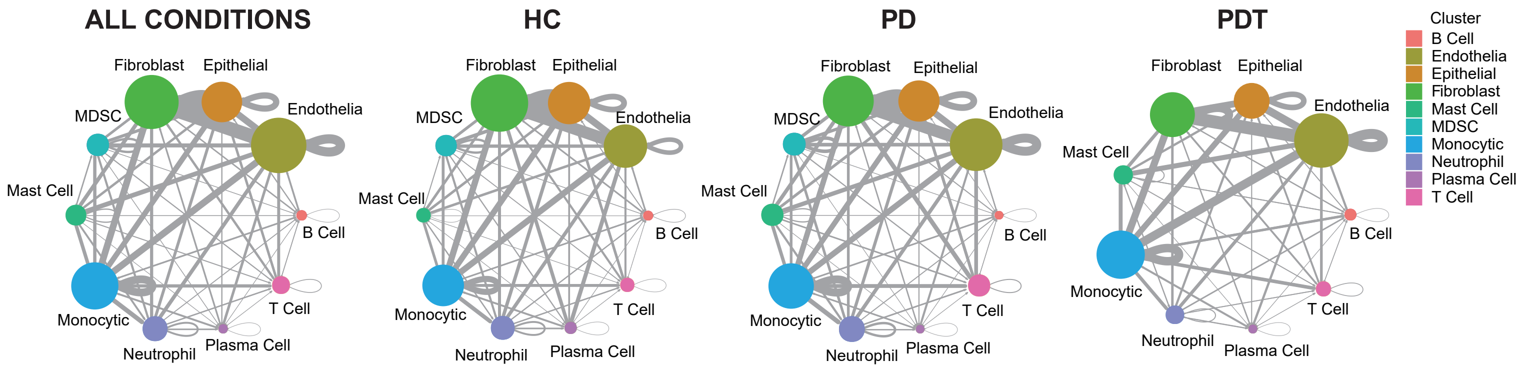

Figure S10. The circo plots under distinct conditions related to Figure 7A

The circo plots showing the potential cell interactions among ten major cell types as in Figure 1B in periodontal tissues predicted by Cellphone DB, from left to right: all conditions, HC, PD and PDT. The node size represents the number of interactions.

Figure S11

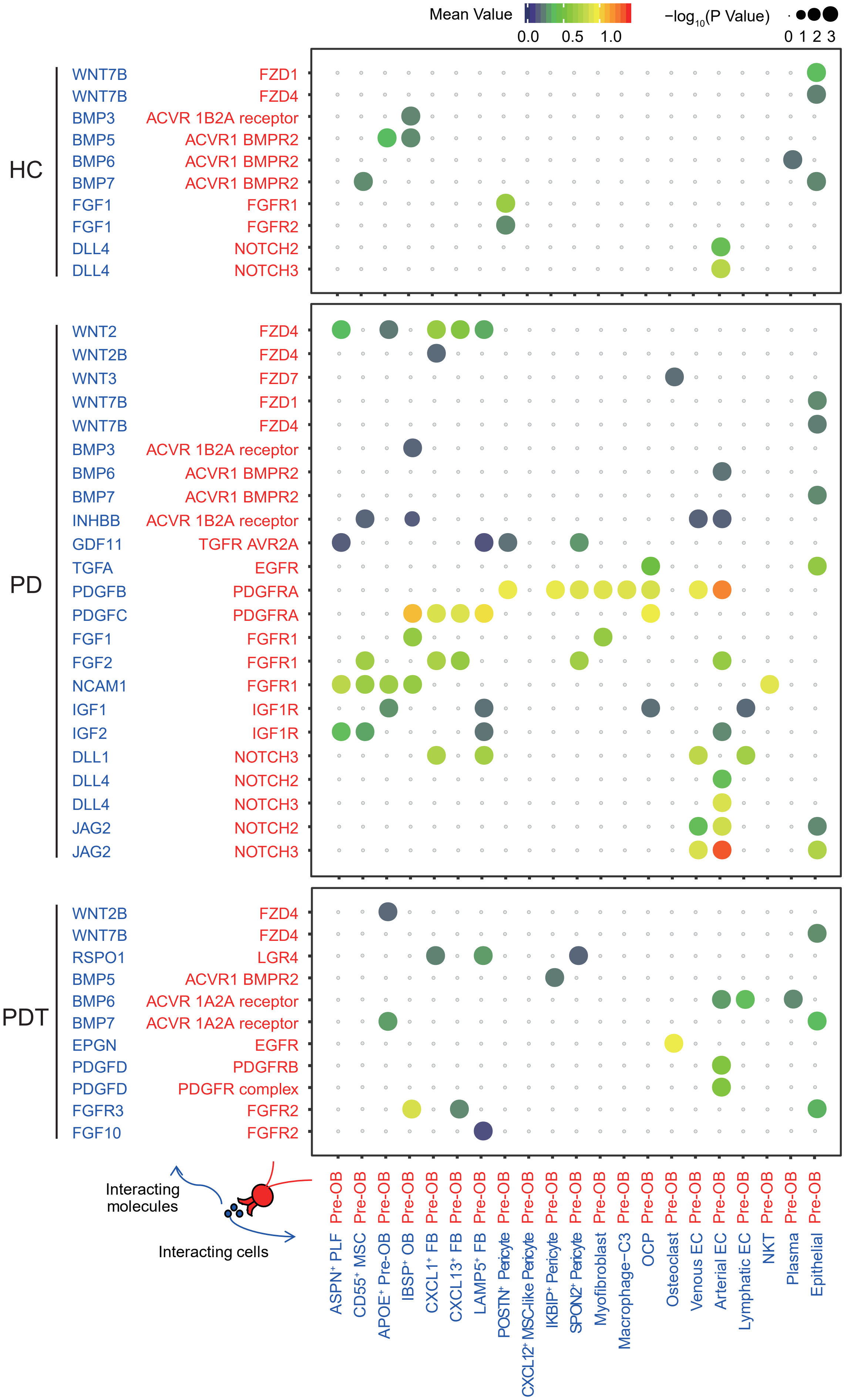

Figure S11. Expression of ligand-receptor pairs between Pre-OB and all cell types in three sample sets, related to Figure 7C

The dot plot generated by CellPhoneDB showing potential ligand-receptor pairs associated with osteogenesis between Pre-OB and all detected cellular types in HC, PD, and PDT groups (from top to bottom). Dots colored by mean expression of ligand-receptor pair between two clusters and dots size proportional to the value of -$\log_{10}$(P Value).

Figure S12

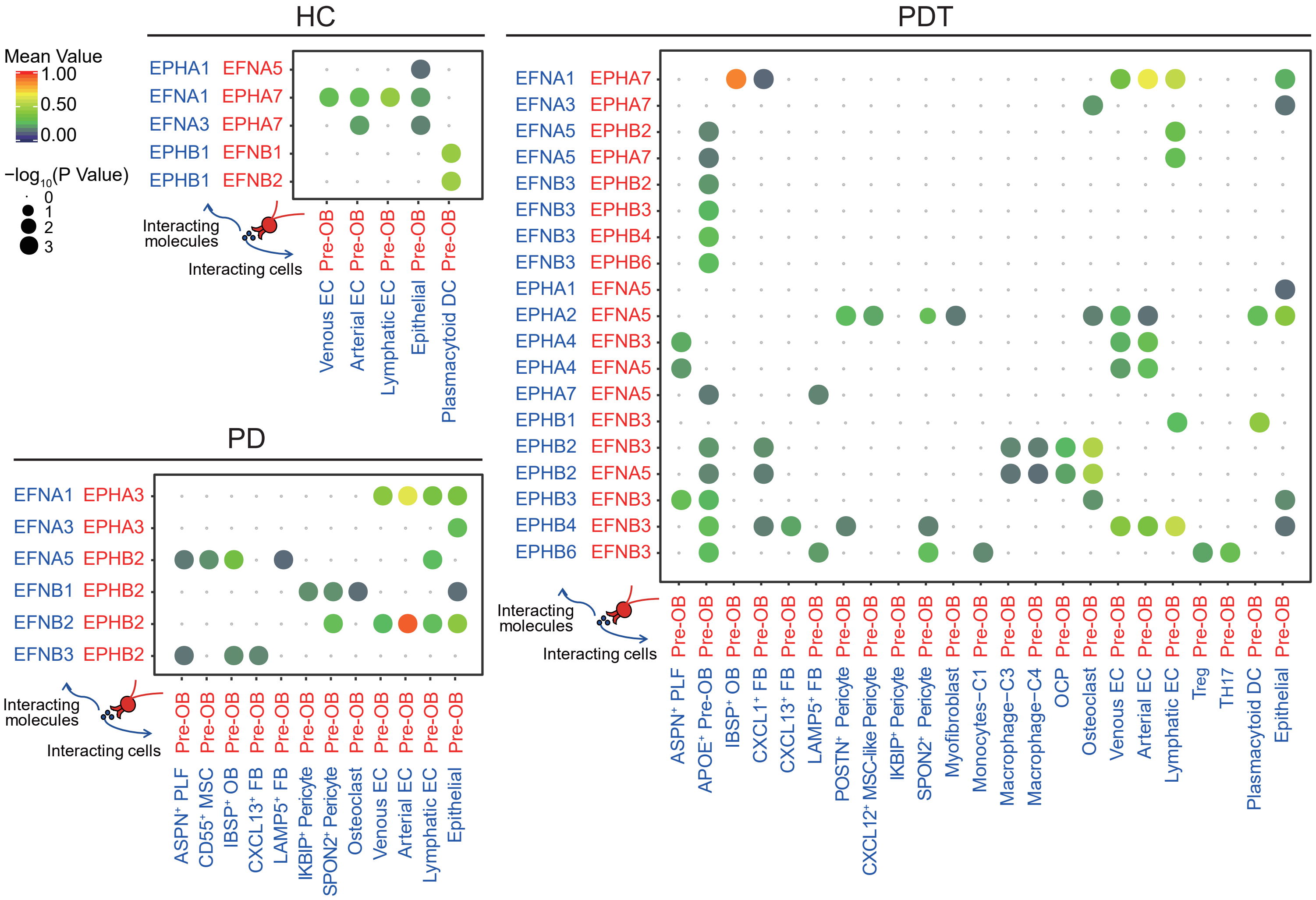

Figure S12. Expression of Ephrin-Ephn interactions between Pre-OB and all cell types in three sample sets, related to Figure 7E

The dot plots generated by CellPhoneDB showing potential ligand-receptor pairs associated with Ephrin-Ephn signaling pathway between Pre-OB and all detected cellular types in HC (top left), PD (bottom left), and PDT (right) groups. Dots colored by mean expression of ligand-receptor pair between two clusters and dots size proportional to the value of $-\log_{10}$ (P Value).

Figure S13

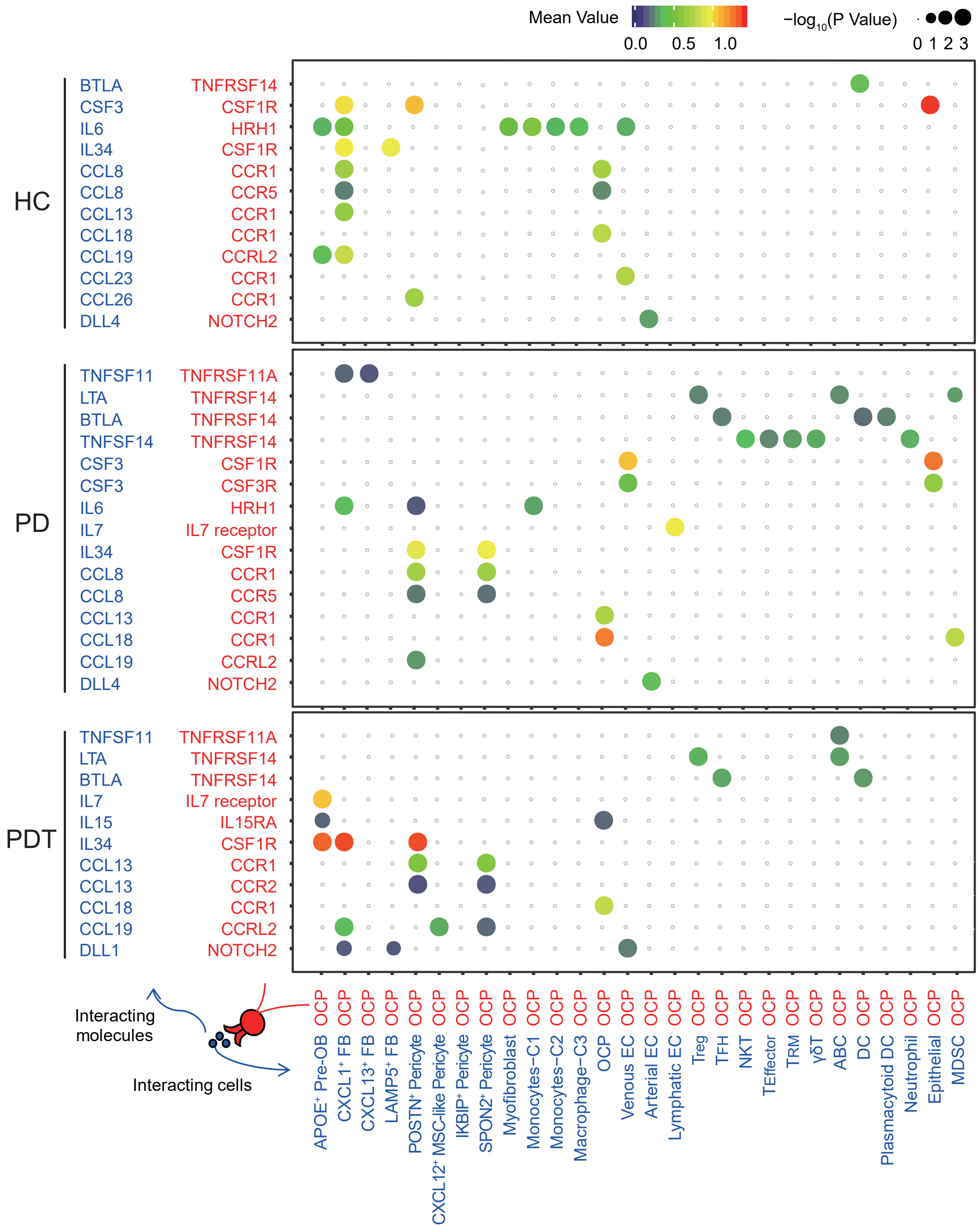

Figure S13. Expression of ligand-receptor pairs associated between OCP and all cell types in three sample sets

The dot plot generated by CellPhoneDB showing potential ligand-receptor pairs associated with osteoclastogenesis between OCP and all detected cellular types in HC, PD, and PDT groups (from top to bottom). Dots colored by mean expression of ligand-receptor pair between two clusters and dots size proportional to the value of -log$_{10}$ (P Value).

Figure S14

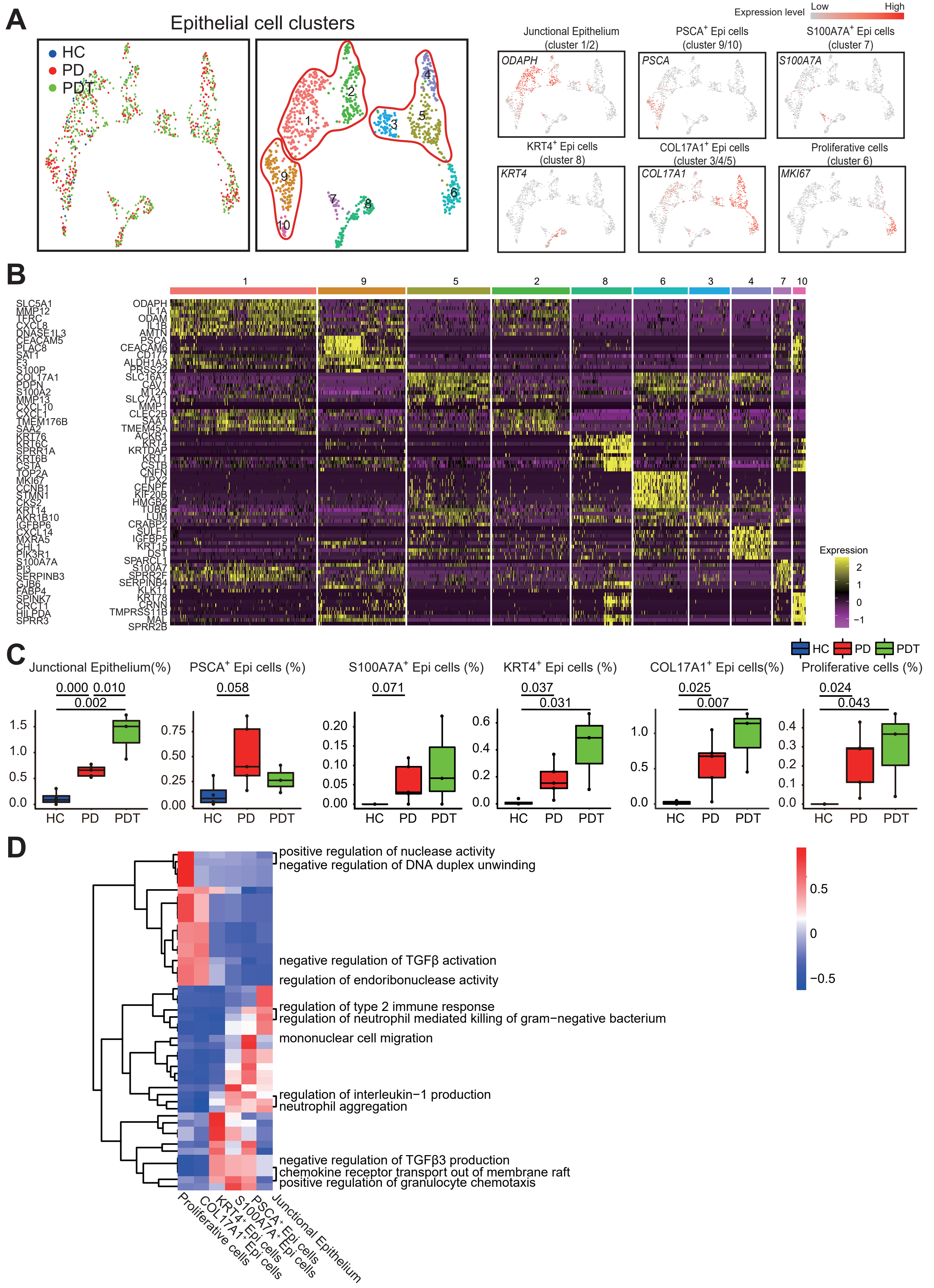
A
Epithelial cell clusters
HC
PD
PDT
Expression level
Low
High
Junctional Epithelium (cluster 1/2)
ODAPH
PSCA⁺ Epi cells (cluster 9/10)
PSCA
S100A7A⁺ Epi cells (cluster 7)
S100A7A
KRT4⁺ Epi cells (cluster 8)
KRT4
COL17A1⁺ Epi cells (cluster 3/4/5)
COL17A1
Proliferative cells (cluster 6)
MKI67
B
Expression
C
Junctional Epithelium(%)
PSCA⁺ Epi cells (%)
S100A7A⁺ Epi cells (%)
KRT4⁺ Epi cells (%)
COL17A1⁺ Epi cells(%)
Proliferative cells (%)
0.000
0.010
0.002
0.058
0.071
0.037
0.031
0.025
0.007
0.024
0.043
D
positive regulation of nuclease activity
negative regulation of DNA duplex unwinding
negative regulation of TGFβ activation
regulation of endoribonuclease activity
regulation of type 2 immune response
regulation of neutrophil mediated killing of gram−negative bacterium
mononuclear cell migration
regulation of interleukin−1 production
neutrophil aggregation
negative regulation of TGFβ3 production
chemokine receptor transport out of membrane raft
positive regulation of granulocyte chemotaxis
Proliferative cells
COL17A1⁺ Epi cells
KRT4⁺ Epi cells
S100A7A⁺ Epi cells
PSCA⁺ Epi cells
Junctional Epithelium

Figure S14. Identification of distinct clusters and functions in epithelial cell population

(A) Left panels: Uniform Manifold Approximation and Projection (UMAP) of 1029 epithelial cells (as in Figure 1A), annotated and colored by the sample type of origin (HCs, PDs, and PDTs) and clustering. Right panels: UMAPs color-coded for expression (gray to red) of key cell-type markers to define the clusters. Red contours (from left to right): PSCA$^+$ Epi cells (cluster 9/10); Junctional Epithelium (cluster 1/2); COL17A1$^+$ Epi cells (cluster 3/4/5). Epi cells: Epithelial cells.
(B) Heatmap of the relative expression level of the top 8-10 differentially expressed maker genes identified in the fibroblast clusters in (A).
(C) The box plots showing the percentage of cells for each of six clusters as in (A) from HC (blue, n=37), PD (red, n=449), and PDT samples (green, n=543) with plot center, box, whiskers, and points corresponding to the median, IQR, 1.5 × IQR and >1.5× IQR, respectively. Data were analyzed using t-tests (two-tailed, two sample, equal variance).
(D) Heatmap of the QuSAGE activity for GO terms that are associated with differentially expressed marker genes among six clusters (labels on the bottom). Red indicating increased average expression of genes in GO term. Epi cells: Epithelial cells.
